# Niche stiffness sustains cancer stemness via TAZ and NANOG phase separation

**DOI:** 10.1038/s41467-023-35856-y

**Published:** 2023-01-16

**Authors:** Xinwei Liu, Yingying Ye, Liling Zhu, Xiaoyun Xiao, Boxuan Zhou, Yuanting Gu, Hang Si, Huixin Liang, Mingzhu Liu, Jiaqian Li, Qiongchao Jiang, Jiang Li, Shubin Yu, Ruiying Ma, Shicheng Su, Jian-You Liao, Qiyi Zhao

**Affiliations:** 1 Department of Infectious Diseases, Third Affiliated Hospital, Sun Yat-Sen University, Guangzhou, 510630 China; 2 Department of Breast Surgery, the First Affiliated Hospital, Zhengzhou University, Zhengzhou, 450000 China; 3 Guangdong Key Laboratory of Liver Disease Research, Third Affiliated Hospital, Sun Yat-sen University, Guangzhou, 510630 China; 4 Guangdong Provincial Key Laboratory of Malignant Tumor Epigenetics and Gene Regulation, Medical Research Center, Sun Yat-Sen Memorial Hospital, Sun Yat-Sen University, Guangzhou, 510120 China; 5 Breast Tumor Center, Sun Yat-Sen Memorial Hospital, Sun Yat-Sen University, Guangzhou, 510120 China; 6 Department of Ultrasound, Sun Yat-Sen Memorial Hospital, Sun Yat-Sen University, Guangzhou, 510120 China; 7 Department of Immunology, Zhongshan School of Medicine, Sun Yat-Sen University, Guangzhou, 510080 China

**Keywords:** Breast cancer, Cancer microenvironment, Cancer stem cells

## Abstract

Emerging evidence shows that the biomechanical environment is required to support cancer stem cells (CSCs), which play a crucial role in drug resistance. However, how mechanotransduction signals regulate CSCs and its clinical significance has remained unclear. Using clinical-practice ultrasound elastography for patients’ lesions and atomic force microscopy for surgical samples, we reveal that increased matrix stiffness is associated with poor responses to neoadjuvant chemotherapy, worse prognosis, and CSC enrichment in patients with breast cancer. Mechanically, TAZ activated by biomechanics enhances CSC properties via phase separation with NANOG. TAZ-NANOG phase separation, which is dependent on acidic residues in the N-terminal activation domain of NANOG, promotes the transcription of SOX2 and OCT4. Therapeutically, targeting NANOG or TAZ reduces CSCs and enhances the chemosensitivity in vivo. Collectively, this study demonstrated that the phase separation of a pluripotency transcription factor links mechanical cues in the niche to the fate of CSCs.

## Abstract (web summary)

Stromal cells in the tumour microenvironment are reported to regulate cancer stemness via biomechanical signals. Here the authors show that TAZ activated by biomechanical cues enhanced cancer stem cell properties via phase separation with Nanog.

## Introduction

Tissue force and mechanical signals in the niche program the fate of stem and progenitor cells, and play a critical role in tissue development and homeostasis^1,2^. Multipotent stem cells are tightly regulated by mechanical force for a dynamic control of their self-renewal and differentiation that are required for sustaining normal structure and function^3^. Deficiency in tensional homeostasis may alter cells fate to initiate cell transformation and facilitate the later development of tumorigenic lesions^2,4,5^. Mechanically responsive sensors responding to extracellular biomechanical force are coupled with intracellular biochemical signaling pathways, to then regulate cell behaviors^6^.

Cancer stem cells (CSCs) exhibit the properties of self-renewal and play a pivotal role in tumor initiation and drug resistance^4,7^. Similar to stem cells, CSCs also reside in a niche, which maintains the principal properties of CSCs, preserves their phenotypic plasticity, and protects them from drugs and immune response^8–10^. Chen et al.^11–13^ and previous studies^4,8^ have shown that the stromal cells in the niche induce and maintain CSC stemness and chemoresistance via biochemical factors, including cytokines, membrane proteins, and non-coding RNAs in extracellular vesicles. Recent evidence suggests that mechanical disruption plays an important role in regulating cancer stemness^14,15^. Transcriptional coactivator TAZ of the Hippo pathway is an important mechanotransducer associated with the pathogenesis of multiple diseases, including cancer^16^. However, direct clinical evidence on whether the mechanical niche of CSCs drives tumor progression in patients is still lacking. More importantly, the underlying mechanism of how niche stiffness mediates the properties of CSCs is largely unclear.

The use of neoadjuvant (preoperative) systemic therapy is currently increasing because it facilitates breast-conserving surgery and the measurement of in vivo responses to systemic treatment^17^. Moreover, previous studies^11,12,18–22^ reported neoadjuvant chemotherapy (NAC) as an ideal clinical evaluation model for the intra-patient comparison of tumor microenvironment changes during treatment, because paired pre-treatment diagnostic biopsies and post-treatment surgically resected samples could be obtained from the same patients. Using clinical-practice ultrasound elastography (UE) for patients’ lesions and atomic force microscopy (AFM) for surgical specimens, the matrix stiffness at the tissue and cellular levels could be measured, respectively^23,24^.

In the current study, we evaluate the clinical significance of matrix stiffness in breast cancer patients who received NAC, and explore its underlying molecular mechanisms in vitro and in a mouse model in vivo.

## Results

### Matrix stiffness is correlated with chemoresistance and poor survival in patients with breast cancer

**Fig. 1:**
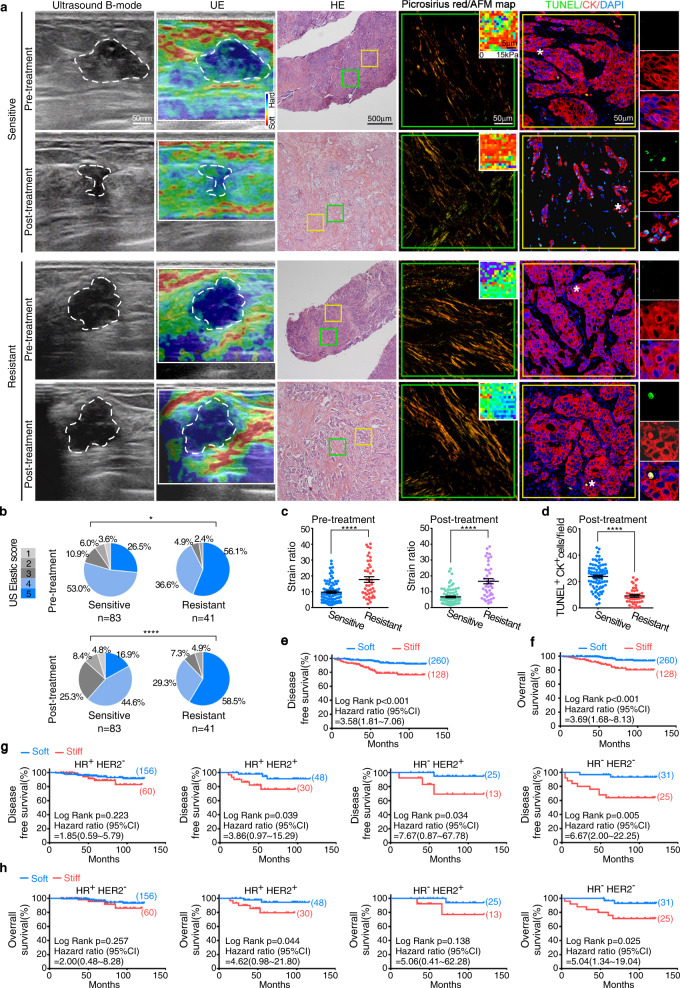
Matrix stiffness is correlated with chemoresistance and poor survival in patients with breast cancer. **a** The lesions of patients with breast cancer in the NAC cohort were evaluated by UE before and after neoadjuvant chemotherapy. Additionally, the pre-treatment biopsies and paired post-treatment resected samples were assessed by HE staining, picrosirius red staining, AFM, and immunofluorescence analysis. Representative images of ultrasound B-mode, UE, HE, picrosirius red staining, AFM map (inserted in picrosirius red pictures), and TUNEL/CK staining are shown (*n* = 124 patients). White dashed lines represent tumor outlines. Green and yellow squares in the HE pictures indicate the area shown in the picrosirius red and immunofluorescence pictures, respectively. Asterisks (*) indicate location of higher magnification views in TUNEL/CK staining images. Picrosirius red staining viewed under polarized light taken from breast cancer tissues reveal the fibrillar collagen in the ECM; AFM indentations are represented as a force heat map. **b** Quantification of tumor elastic scores in patients as (**a**) in the NAC cohort. Patients with CR or PR were classified as chemosensitive patients (*n* = 83 patients), while those with SD or PD were classified as chemoresistant ones (*n* = 41 patients). *p = 0.0237, ****p < 0.0001, by unpaired two-tailed Fisher exact probability test. **c** Quantification of tumor strain ratio in patients as (**a**). Mean ± SEM, ****p < 0.0001 by two-tailed Mann-Whitney U test. **d** Quantification of apoptotic tumor cells (TUNEL^+^CK^+^) in breast cancer samples obtained from patients of post-neoadjuvant chemotherapy. *N* = 83 for chemosensitive patients and *n* = 41 for chemoresistant patients. Mean ± SEM, ****p < 0.0001 by two-tailed Mann-Whitney U test. **e**, **f** Kaplan-Meier curves for DFS or OS of breast cancer patients with soft (strain ratio ≤ 8.7, *n* = 260 patients) or stiff (strain ratio > 8.7, *n* = 128 patients) matrix in the treatment-naive cohort. **g**, **h** The Kaplan–Meier plot for DFS (**g**) and OS (**h**) of patients with different subtypes in the treatment-naive cohort. HR^+^HER2^-^, *n* = 216 patients; HR^+^HER2^+^, *n* = 78 patients; HR^-^HER2^+^, *n* = 38 patients; HR^-^HER2^-^, *n* = 56 patients. For **e**–**h** *P* values were determined by log-rank test and shown in the figure.

Matrix stiffness could modulate the chemosensitivity of cancer cells in vitro and in animals^25–27^; however, the clinical significance in therapeutic response and long-term prognosis of breast cancer patients remains poorly understood^28^. Thus, we employed the NAC clinical model^17^, wherein tumor shrinkage and tumor biology could be monitored during treatment, to evaluate the clinical significance of matrix stiffness in breast cancer. We measured the elastic score and strain ratio of lesions in 124 female patients with breast cancer (Supplementary Table 1) by UE before and after NAC (Fig. 1a-c). The tumor elastic scores of the 41 chemoresistant patients with progressive disease (PD) or stable disease (SD) were markedly higher than those of the 83 chemosensitive patients with complete remission (CR) or partial remission (PR), both before and after NAC (Fig. 1a, b). Furthermore, strain ratio measurement showed similar results (Fig. 1a, c). To validate the results obtained by UE at the cellular level, we evaluated extracellular matrix (ECM) stiffness in paired pre-treatment biopsy and post-treatment resected samples from the same patients by AFM (Fig. 1a). Picrosirius red staining revealing collagen fibrils in ECM and AFM indentations were performed in areas corresponding to ECM. Consistently, the ECM of both pre-treatment and post-treatment samples from the chemoresistant patients exhibited higher Young’s modulus compared with those of chemosensitive patients (Fig. 1a and Supplementary Fig. 1a). Furthermore, fewer apoptotic cancer cells (TUNEL^+^CK^+^) were found in the residual tissues with higher matrix stiffness after NAC (Fig. 1a, d). Together, these data suggested that increased matrix stiffness is associated with poor chemotherapeutic response. To investigate whether matrix stiffness is a prognostic factor, we used X-tile statistical software to determine an optimal cutoff point of 8.7 for the strain ratio using a minimal p-value approach in 388 treatment-naive breast carcinoma samples (Supplementary Table 2). Higher matrix stiffness was correlated with shorter disease-free survival (DFS) and overall survival (OS) (Fig. 1e, f). Furthermore, multivariate Cox regression analysis showed matrix stiffness as an independent prognostic factor for DFS (Table 1) and OS (Table 2) after adjusting for other variables associated with the patients’ prognosis, including tumor size, lymph node status, histological grading, and estrogen receptor (ER) status. The 388 patients were further divided into 4 molecular subtypes according to the hormone receptor (HR) and HER2 status. Of note, low matrix stiffness in the triple-negative subtype (HR^-^HER2^-^) of breast cancer was significantly associated with better survival (Fig. 1g, h). Collectively, the data presented here demonstrate that matrix stiffness is a clinically measurable prognostic factor for breast cancer patient prognosis.

**Table 1:** Cox regression analysis of disease-free survival in the treatment-naive cohort (*n* = 388)

Factor	Disease-Free Survival			
	Univariable	Multivariable		
	P value	HR	95CI%	p value
Age (>45 years)	0.165			
Premenopause	0.404			
Tumor size (>2 cm)	0.002	2.003	1.014–3.953	0.045*
Lymph node (positive)	0.001	2.145	1.086–4.240	0.028*
Histological grade (III)	0.019	1.526	0.800–2.912	0.199
ER (positive)	0.022	0.504	0.267–0.952	0.035*
PR (positive)	0.318			
HER2 (positive)	0.513			
Strain ratio (>8.7)	<0.001	2.776	1.448–5.322	0.002*

*P* value was calculated with two-sided log-rank test. *p < 0.05. Histological grade was determined according to the Elston-Ellis system; *ER* estrogen receptor, *PR* progesterone receptor, *HER2* human epidermal growth factor receptor 2.

**Table 2:** Cox regression analysis of overall survival in the treatment-naive cohort (*n* = 388)

Factor	Overall Survival			
	Univariable	Multivariable		
	P value	HR	95CI%	p value
Age (>45 years)	0.349			
Premenopause	0.200			
Tumor size (>2 cm)	0.003	2.389	1.103–5.178	0 .027*
Lymph node (positive)	0.002	2.413	1.088–5.351	0.030*
Histological grade (III)	0.431			
ER (positive)	0.140			
PR (positive)	0.062			
HER2 (positive)	0.426			
Strain ratio (>8.7)	<0.001	3.275	1.568–6.840	0.002*

*P* value was calculated with two-sided log-rank test. *p < 0.05. Histological grade was determined according to the Elston-Ellis system; *ER* estrogen receptor, *PR* progesterone receptor, *HER2* human epidermal growth factor receptor 2.

### Matrix stiffness is correlated with CSC abundance in human breast cancer

**Fig. 2:**
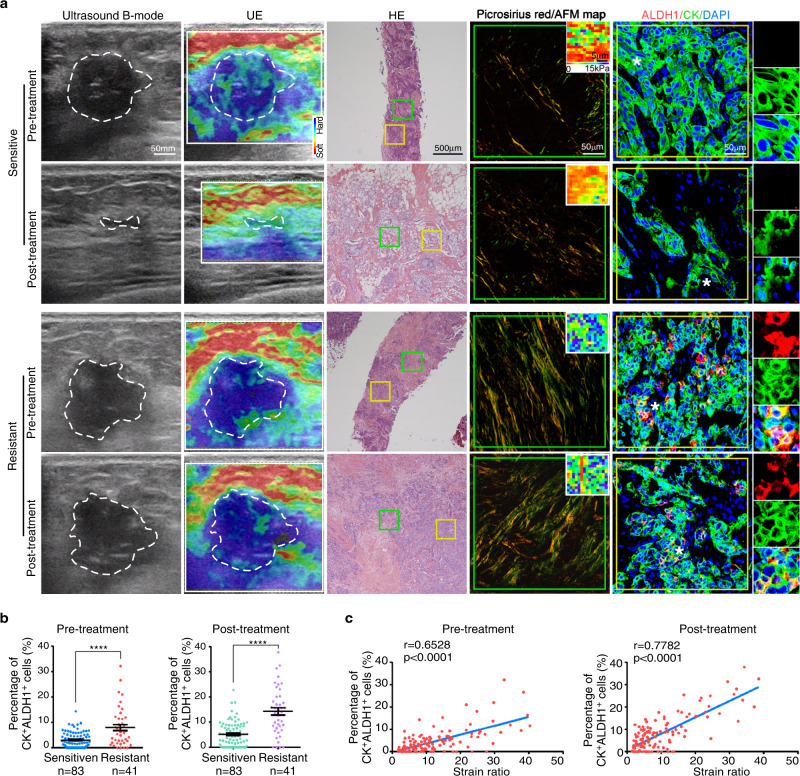
Matrix stiffness is correlated with CSC abundance in human breast cancer. **a** Representative images of ultrasound B-mode, UE, HE, picrosirius red staining, AFM map (inserted in picrosirius red pictures), and ALDH1/CK staining of patients in the NAC cohort (*n* = 124 patients). White dashed lines represent tumor outlines. Green and yellow squares in the HE pictures indicate the area shown in the picrosirius red and immunofluorescence pictures, respectively. Asterisks (*) indicate location of higher magnification views in ALDH1/CK staining images. Bars in the pictures indicate the scale. **b** Quantitation of CK^+^ALDH1^+^ CSCs percentage in total cancer cells of patients in **(a)** (*n* = 124 patients). Mean ± SEM, ****p < 0.0001 by two-tailed Mann-Whitney U test. **c** The correlation between the strain ratio and the percentage of CK^+^ALDH1^+^ CSCs of patients in **(a)** was evaluated by Pearson’s correlation analysis (*n* = 124 patients). Correlation coefficient (r) and p value were acquired by Pearson correlation test and shown in the figure. For **b** and **c**, source data are provided as a Source Data file.

CSCs were shown to be a key population of tumor cells playing a pivotal role in chemoresistance^4,8,11–13^. To investigate whether high matrix stiffness is associated with CSC enrichment in patients, we evaluated CSCs by double immunostaining for acetaldehyde dehydrogenase 1 (ALDH1) and cytokeratin (CK). ALDH1 is a marker of CSCs that can reduce the effects of toxic aldehydes on cancer cells leading to cancer progression, self-protection, and self-renewal^29^. The proportions of ALDH1^+^CK^+^ CSCs were higher in the chemoresistant samples than in the sensitive ones in biopsies obtained prior to NAC and resected samples after NAC (Fig. 2a, b). More importantly, the abundance of ALDH1^+^CK^+^ tumor cells was significantly correlated with matrix stiffness in both the pre- and post-treatment samples (Fig. 2c). Together, these results indicated that CSCs are enriched in rigid samples of breast cancer patients.

### Matrix stiffness induces chemoresistance and enriches CSCs in breast cancer cells

**Fig. 3:**
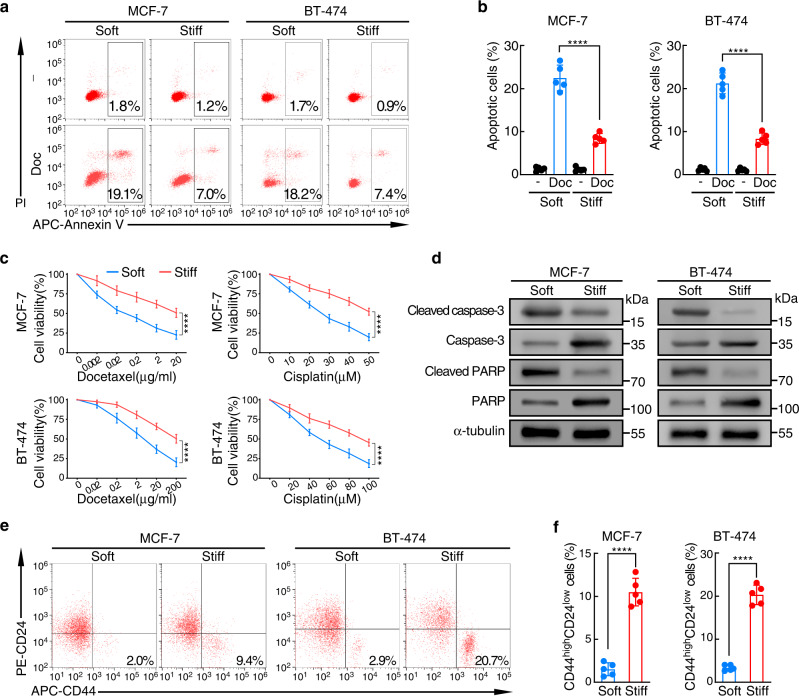
Matrix stiffness induces chemoresistance and enriches CSCs in breast cancer cells. MCF-7 and BT-474 cells were cultured on soft (0.5 kPa) or stiff (9 kPa) supports for 2 weeks before subsequent analysis. **a** Docetaxel (Doc) induced apoptosis of MCF-7 and BT-474 was assessed by flow cytometry. The proportions of Annexin V^+^ cells are shown. **b** Quantitation of apoptotic cells (Annexin V^+^) in MCF-7 and BT-474 cells in **(a)** (*n* = 5 biologically independent experiments). Mean ± SD, ****p < 0.0001 by two-sided two-way ANOVA with Tukey test. **c** MCF-7 and BT-474 cells cultured on soft or stiff supports were treated with docetaxel or cisplatin. Cell viability was evaluated after 24 h by CCK8 assays (*n* = 5 biologically independent experiments). Mean ± SD, ****p < 0.0001 by two-sided Student’s t test. **d** Western blots for cleaved/uncleaved caspase-3 and PARP in docetaxel-treated MCF-7 and BT-474 cells cultured on soft or stiff supports. Representative images from 3 biologically independent experiments are shown. **e** The percentage of CD44^high^CD24^low^ cells in MCF-7 and BT-474 cells cultured on soft or stiff supports were determined by flow cytometry. **f** Quantitation of CD44^high^CD24^low^ cells in **(e)** (*n* = 5 biologically independent experiments). Mean ± SD, ****p < 0.0001 by two-sided Student’s t test. For **b-d** and **f**, source data are provided as a Source Data file.

Given that matrix stiffness in human breast cancer is associated with poor chemotherapeutic response, we investigated whether tumor cells exhibit different chemosensitivities in distinct tensional cultures in vitro. Tumor cells were grown either on a soft polyacrylamide gel (0.5 kPa; matching the compliance of normal mammary gland)^30^, or on a stiff polyacrylamide gel (9 kPa)^31^. Docetaxel- or cisplatin- treated MCF-7 and BT-474 breast cancer cells grown on stiff supports significantly reduced apoptosis compared with those grown on soft supports (Fig. 3a-c and Supplementary Fig. 2a). In agreement, caspase-3 and poly ADP-ribose polymerase (PARP) cleavage were decreased in tumor cells cultured on stiff supports (Fig. 3d).

Next, given that more ALDH1^+^ tumor cells were observed in stiffer clinical samples, we asked whether the cancer stemness of culture cells was also affected by matrix stiffness. Consistent with the chemosensitivity experiment results, breast CSC proportions were markedly increased in both MCF-7 and BT-474 cancer cells grown on stiff supports (Fig. 3e, f and Supplementary Fig. 2b-d). Together, these data suggested that matrix stiffness induces chemoresistance and enriches CSCs in breast cancer cells.

### TAZ regulates chemoresistance and cancer stemness in vitro

**Fig. 4:**
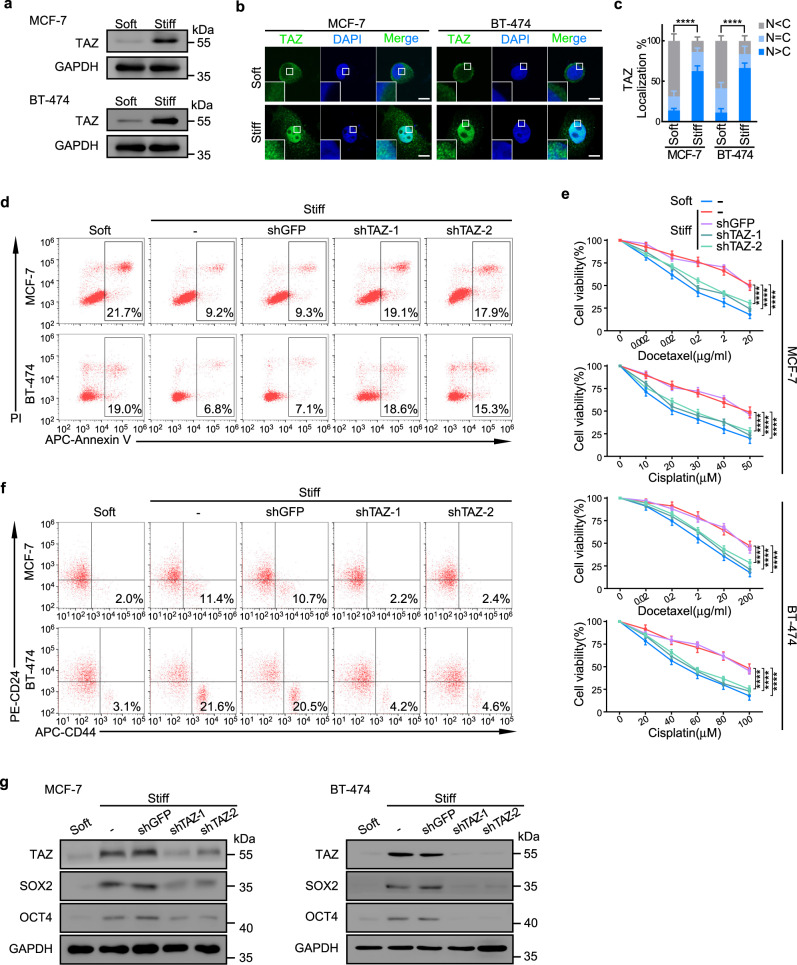
TAZ regulates chemoresistance and cancer stemness in vitro. MCF-7 and BT-474 cells were cultured on soft or stiff supports for 2 weeks before subsequent analysis. In some experiments, cells were pre-transduced with TAZ shRNA (shTAZ-1 or shTAZ-2) or GFP shRNA (shGFP). **a** The expression of TAZ in MCF-7 and BT-474 cells cultured on soft or stiff supports was determined by western blot (*n* = 3 biologically independent experiments). **b** Representative immunofluorescence staining of TAZ in MCF-7 and BT-474 cells cultured on soft or stiff substrates. The insert represents a magnification from the indicated area (white square). Scale bars, 10 μm. **c** Proportion of cancer cells exhibiting mainly nuclear TAZ localization (N > C), diffuse distribution of TAZ in nucleus and cytoplasm (N = C), or preferential cytoplasmic TAZ (N < C or undetectable). 20 cells were assessed per group, experiments were independently repeated 4 times. Mean ± SD, ****p < 0.0001 by two-tailed chi-square test. **d** Docetaxel induced apoptosis of MCF-7 and BT-474 was assessed by flow cytometry. See Supplementary Fig. 3a for quantification. **e** MCF-7 and BT-474 cells cultured on soft or stiff supports were treated with docetaxel or cisplatin. Cell viability was evaluated by CCK8 assay (*n* = 5 biologically independent experiments). Mean ± SD, ****p < 0.0001 by two-sided one-way ANOVA with Tukey test. **f** Representative images of CD44^high^CD24^low^ cells in MCF-7 and BT-474 cells were detected by flow cytometry. See Supplementary Fig. 3b for quantification. **g** Western blots of TAZ, SOX2, and OCT4 in MCF-7 and BT-474 cells cultured on soft or stiff supports (*n* = 3 biologically independent experiments). For data presented in the same figure panel, the samples were derived from the same experiment and blots were processed in parallel. For **a**, **c**, **e** and **g**, source data are provided as a Source Data file.

TAZ is a key mechanotransducer that regulates CSCs^32^, albeit the underlying mechanism remains unclear. Consistently, we found that TAZ levels were upregulated (Fig. 4a) and accumulated in the nuclei (Fig. 4b, c) of MCF-7 and BT-474 cells grown on stiff supports compared with soft supports.

To investigate whether TAZ regulated stiffness-dependent apoptosis and stemness, we silenced endogenous TAZ with shRNAs in MCF-7 and BT-474 cells grown on stiff supports and observed that apoptosis rates were restored to a comparable level of cancer cells grown on soft supports (Fig. 4d and Supplementary Fig. 3a). Furthermore, when challenged with docetaxel or cisplatin on stiff supports, MCF-7 and BT-474 cells with TAZ knockdown exhibited markedly reduced cell viability (Fig. 4e) and CSC proportions (Fig. 4f and Supplementary Fig. 3b).

Moreover, stem cell-associated transcription factors SOX2 and OCT4 were upregulated in MCF-7 and BT-474 cells cultured on stiff supports compared to those grown on soft supports (Fig. 4g). Silencing TAZ in tumor cells cultured on stiff supports markedly reduced SOX2 and OCT4 expression (Fig. 4g), thus indicating that TAZ regulates the stiffness-dependent cancer stemness of breast cancer cells.

### TAZ regulates SOX2 and OCT4 expression through phase separation with NANOG

**Fig. 5:**
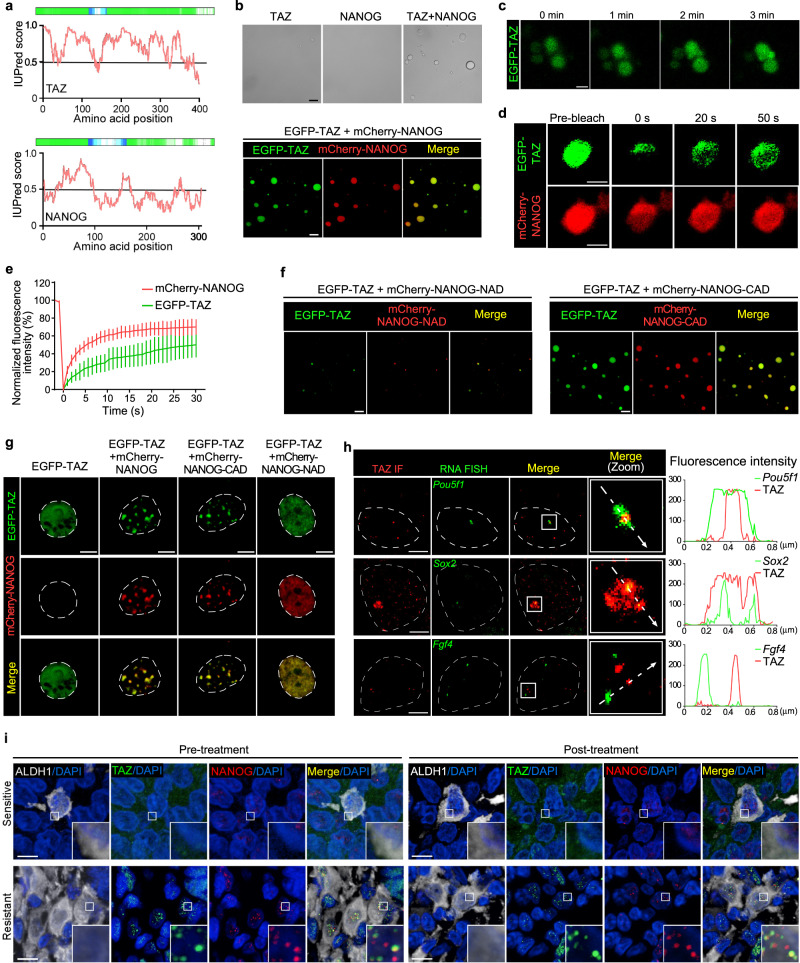
TAZ regulates SOX2 and OCT4 expression through phase separation with NANOG. **a** The intrinsic disorder tendency of TAZ and NANOG was predicted by IUPred and D_2_P_2_ algorithms. A score above 0.5 indicates disorder. **b** Top, solutions containing the indicated protein at 10 μΜ (ΤΑΖ and NANOG) were examined by phase contrast microscopy. Bottom, the indicated EGFP-TAZ and mCherry-NANOG fusion proteins were mixed at 10 μM, examined by confocal microscopy at room temperature. Representative image from 3 biologically independent experiments. Scale bar, 10 μm. **c** EGFP-TAZ (10 µM) was mixed with mCherry-NANOG (10 µM) and imaged by confocal microscopy. The fusion of EGFP-TAZ droplets was shown (*n* = 3 biologically independent experiments). Scale bar, 2 μm. **d**, **e**, **g**, **h** HEK293T cells were transfected with EGFP-TAZ, mCherry-NANOG, mCherry-NANOG-CAD mutation, or mCherry-NANOG-NAD mutation. **d**, Representative live cell images of the fluorescence recovery after photobleaching (FRAP) experiment are shown. Scale bar, 2 μm. **e**, Quantification of FRAP analysis in **(d)**. 0 s is the starting of the bleaching event. Plots were generated from 20 droplets in 4 independent experiments. Mean ± SD. **f** Confocal images of recombinant EGFP-TAZ (10 µM) in the presence of mCherry-NANOG-NAD or mCherry-NANOG-CAD are shown. Scale bars, 10 μm. The image is representative of three independent experiments. **g** Confocal microscopy images of HEK293T cells transfected with EGFP-TAZ and mCherry-NANOG/ mCherry-NANOG-CAD/ mCherry-NANOG-NAD. White dashed lines denote the contours of the cell nucleus. Scale bars, 5 μm. See Supplementary Fig. 4b for quantification. **h** Confocal images of TAZ immunofluorescence and *Pou5f1*, *Sox2*, and *Fgf4* loci by concurrent nascent RNA-FISH in HEK293T cells. White dashed lines denote the contours of the cell nucleus. Merge (zoom) represents a zoomed-in view of the white box. Line plot analyzed the colocalization of RNA FISH foci and TAZ puncta. Scale bar, 2 μm. See Supplementary Fig. 4c for quantification. **i** Representative images (maximal projections in z) of ALDH1, TAZ, NANOG and DAPI immunofluorescence staining in paired samples of breast cancer patients with sensitive or resistant responses before and after NAC. White box indicates the areas enlarged in the insets. Scale bar, 10 μm. See Supplementary Fig. 4k for quantification.

NANOG, a master transcriptional regulator of stemness, is linked to cancer progression and chemoresistance^33,34^. Recently, multiple transcriptional coactivators, including TAZ have been shown to activate genes via the phase separation capacity of their activation domains^35,36^. We found that both TAZ and NANOG contain intrinsically disordered regions (IDRs) (Fig. 5a), which are crucial for the generation of biomolecular condensates. We mixed purified TAZ and NANOG proteins and observed that droplets of EGFP-TAZ incorporated and concentrated mCherry-NANOG to form larger and more numerous droplets (Fig. 5b). The TAZ-NANOG spheres displayed liquid-like condensate characteristics, including droplet coalescence (Fig. 5c) and fast fluorescence recovery after photobleaching (Fig. 5d, e). In line with the in vitro assays, MCF-7 and BT-474 cells cultured on stiff supports caused increased amounts of the TAZ and NANOG protein in the pellet fraction isolated from cells in sedimentation assay (Supplementary Fig. 4a).

Transcription factors could regulate transcriptional output through their activation domains enriched in acidic amino acids to form phase-separated condensates with their coactivator^37^. We therefore generated two NANOG-acidic mutants in which all acidic residues in the N-terminal activation domain (NANOG-NAD) or C-terminal activation domain (NANOG-CAD) were replaced with alanine. The in vitro droplet-formation assays showed that full-length TAZ failed to form droplets with NANOG-NAD mutant. By contrast, the NANOG-CAD mutant did not affect the TAZ-NANOG droplet formation (Fig. 5f). Consistent with the in vitro assays, the NANOG-NAD mutant but not NANOG-CAD mutant significantly impaired the ability of TAZ to form nuclear puncta in HEK293T cells (Fig. 5g and Supplementary Fig. 4b). These results indicate that the formation ability of phase-separated TAZ-NANOG droplets is dependent on acidic residues in the N-terminal activation domain.

We next examined whether TAZ-NANOG phase separation regulates SOX2 and OCT4 expression. Immunofluorescence coupled with concurrent nascent RNA fluorescence in situ hybridization (FISH) revealed that discrete TAZ signals were enriched at the center of the RNA FISH foci of *Sox2* and *Pou5f1* (encoding OCT4) (Fig. 5h). This enrichment was not observed using randomly selected RNA FISH probes for *Fgf4* (Fig. 5h and Supplementary Fig. 4c). Furthermore, the NANOG-NAD mutant, which impaired TAZ-NANOG phase separation, significantly decreased the SOX2 and OCT4 transcriptions (Supplementary Fig. 4d). Interestingly, the NANOG-NAD or 1,6-hexanediol that impaired TAZ-NANOG phase separation largely reduced ALDH1^+^ CSC proportions and cell viability in MCF-7 and BT-474 cells grown on stiff supports (Supplementary Fig. 4e-j). Clinically, we found that TAZ and NANOG formed nuclear puncta in ALDH1^+^ tumor cells in both pre-treatment and post-treatment samples from the chemoresistant breast cancer patients rather than chemosensitive ones (Fig. 5i and Supplementary Fig. 4k). In addition, a proximity ligation assay (PLA) demonstrated the interaction of TAZ and NANOG in chemoresistant patients (Supplementary Fig. 4l-m).

**Fig. 6:**
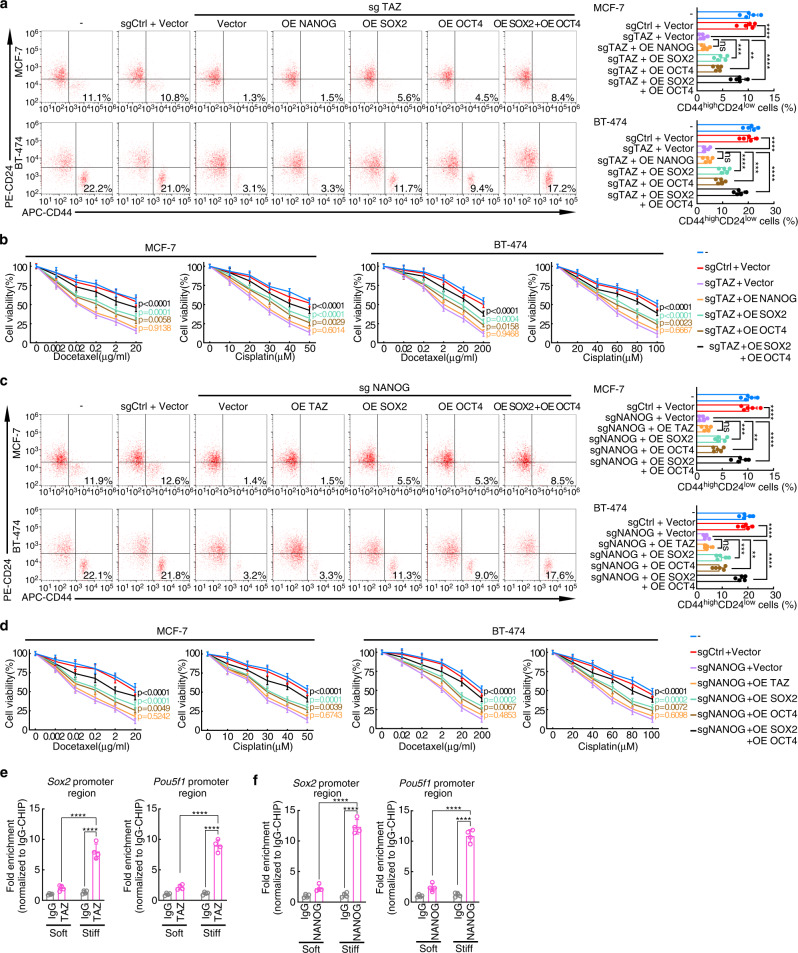
Roles of TAZ and NANOG on SOX2 and OCT4. **a**–**d** TAZ and NANOG were knockout or overexpressed by lentiviral transfection in MCF-7 and BT-474 cells. NANOG, TAZ, SOX2 or/and OCT4 were overexpressed in TAZ or NANOG knockout cells. Transfected cells were cultured on stiff hydrogels for 2 weeks. OE, overexpression. **a** (Left) Representative flow cytometric plot indicating the percentages of CD44^high^CD24^low^ cells in MCF-7 and BT-474 cells. (Right) Qualification of five biologically independent experiments. ****p < 0.0001; for MCF-7 cells, ***p = 0.0003, **p = 0.0039, ns=0.9899; for BT-474 cells, ***p = 0.0008, ns=0.9982. **b** Cell viability of MCF-7 and BT-474 cells treated with various concentrations of docetaxel and cisplatin for 24 h was determined by CCK8 assays (*n* = 4 biologically independent experiments). P values compared with sgTAZ + Vector group were shown in the figure. **c** (Left) Representative flow cytometric plot indicating the percentages of CD44^high^CD24^low^ cells in MCF-7 and BT-474 cells. (Right) Qualification of five independent experiments. ****p < 0.0001; for MCF-7 cells, ***p = 0.0004, **p = 0.0033, ns=0.9400; for BT-474 cells, ***p = 0.0002, **p = 0.0013, ns=0.9982. **d** Cell viability of MCF-7 and BT-474 cells treated with various concentrations of docetaxel and cisplatin for 24 h was determined by CCK8 assays (*n* = 4 biologically independent experiments). *P* values compared with sgNANOG + Vector group were shown in the figure. For panels **(a–d)**, data are mean ± SD; *p* values were calculated by two-sided one-way ANOVA with Tukey test. **e**, **f** MCF-7 cells were cultured on soft or stiff hydrogels for 2 weeks. Then ChIP was performed using cell lysates with indicated antibodies. **e** The precipitated DNA using IgG and TAZ antibody, respectively, was quantified by qPCR with primers specific to promoter regions of *Sox2* and *Pou5f1*. **f** The precipitated DNA using IgG and NANOG antibody, respectively, was quantified by qPCR with primers specific to promoter regions of *Sox2* and *Pou5f1*. **e**, **f** Fold enrichment was normalized to IgG-ChIP negative control (*n* = 4 biologically independent experiments). Numeric values denote mean ± SD; ****p < 0.0001 by two-sided two-way ANOVA with Tukey test. For **a–f**, source data are provided as a Source Data file.

To confirm the regulations of TAZ and NANOG on SOX2 and OCT4, combined gain and loss of function experiments were performed in MCF-7 and BT-474 cells (Fig. 6a-d and Supplementary Fig. 5). When challenged with chemotherapeutic drugs, MCF-7 and BT-474 cells with TAZ knockout exhibited markedly reduced CD44^high^CD24^low^ CSC proportions and cell viability induced by stiff matrix (Fig. 6a, b and Supplementary Fig. 5). As expected, SOX2 or OCT4 could partially rescue the loss of function of TAZ, and combined SOX2 and OCT4 overexpression could further rescue the effect of TAZ loss of function (Fig. 6a, b and Supplementary Fig. 5). Similarly, NANOG knockout greatly reduced CD44^high^CD24^low^ CSC proportions and cell viability, which could be rescued by SOX2 or/and OCT4 overexpression (Fig. 6c, d and Supplementary Fig. 5). Moreover, the chromatin-immunoprecipitation-quantitative polymerase chain reaction (ChIP-qPCR) assay showed that TAZ and NANOG were enriched at the promoter regions of SOX2 and OCT4 in breast cancer cells grown on stiff supports but not cells grown on soft supports (Fig. 6e, f), thus suggesting that TAZ/NANOG could activate *Sox2* and *Pou5f1* (encoding OCT4) transcription by binding their promoters. Taken together, these data suggest that TAZ and NANOG regulate cancer stemness and chemosensitivity via SOX2 and OCT4. Collectively, these results suggested that the phase separation of NANOG and TAZ promotes the transcription of SOX2 and OCT4.

### TAZ and NANOG regulate cancer stemness and chemoresistance in vivo

**Fig. 7:**
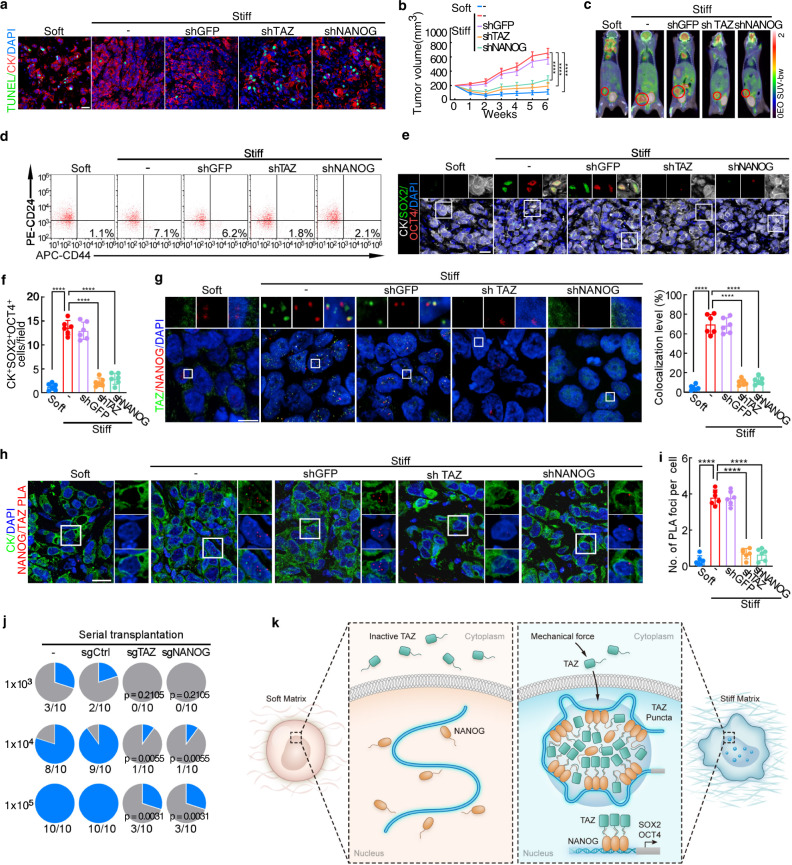
TAZ and NANOG regulate cancer stemness and chemoresistance in vivo. **a–h** MCF-7 cells transduced with shGFP, shTAZ or shNANOG were mixed with hyaluronan-derived hydrogels and injected into the mammary fat pads of NOD/SCID mice. Mice were treated with docetaxel (Doc) per week (*n* = 6 biologically independent animals per group). **a** Representative confocal images of TUNEL and CK in the harvested xenografts. Scale bars, 20 μm. See also Supplementary Fig. 6a. **b** Tumor sizes of the indicated groups. **c** Representative images of tumor growth monitored by PET-CT after six weeks of treatment. Red circles indicate tumors. **d** Tumor cells were isolated from the harvested xenografts. The proportions of CD44^high^CD24^low^ tumor cells were detected by flow cytometry. See also Supplementary Fig. 6b. **e** Representative confocal images for CK, SOX2, and OCT4 in the harvested xenografts. White boxes indicate location of higher magnification views. Scale bars, 20 μm. **f** Quantification of CK^+^SOX2^+^OCT4^+^ cells in Fig.7e. **g** (Left) Representative confocal images (maximal projections in z) for TAZ and NANOG in the xenografts with DAPI (blue) stained nucleus. White box delineates the insets of higher magnification views. Scale bars, 10 μm. (Right) The percentage of NANOG colocalization with TAZ. **h** Proximity ligation assay with TAZ and NANOG antibodies in indicated mice tumor tissues. White boxes indicate area zoomed in and shown in right of images. Scale bars, 20 μm. **i** Quantification of Fig. 7h. For **b**, **f**, **g** and **i**, data are mean ± SD, ****p < 0.0001 by two-sided one-way ANOVA with Tukey test. **j** Incidences of tumorigenesis of the secondary tumor in serial transplantation models of MCF-7 cells are shown (*n* = 10 biologically independent animals per group). P values compared with the first group of each row were calculated by two-sided Fisher’s exact test. **k** Schematic representation of matrix stiffness sustains cancer stemness via TAZ and NANOG phase separation. For **b**, **f**, **g** and **i**, source data are provided as a Source Data file.

To evaluate the therapeutic value of TAZ and NANOG in vivo, we established a mouse xenograft model of breast cancer. Breast cancer cells (MCF-7 and BT-474) were transplanted with hyaluronan-derived hydrogels of different stiffness levels into immunocompromised mice, as previously described^35,38^. The co-transplantation of breast cancer cells with stiff hydrogels (9 kPa), but not soft hydrogels (0.5 kPa), dramatically reduced apoptosis of cancer cells (Fig. 7a and Supplementary Fig. 6a, c) and sustained tumor growth in mice under chemotherapy (Fig. 7b, c and Supplementary Fig. 6d and e). Knockdown of TAZ or NANOG significantly increased apoptosis in tumor cells (Fig. 7a and Supplementary Fig. 6a, c) and suppressed the growth of tumor introduced by mechanical inputs (Fig. 7b, c and Supplementary Fig. 6d and e). Furthermore, stiff matrix increased the proportion of CSCs (Fig. 7d and Supplementary Fig. 6b) and the expression of SOX2 and OCT4, which was abrogated by TAZ or NANOG silencing (Fig. 7e and f). Interestingly, we observed that TAZ mainly dispersed in the cytoplasm of tumor cells injected with soft hydrogels (Fig. 7g and Supplementary Fig. 6f). By contrast, when tumor cells were transplanted with stiff hydrogels into immunocompromised mice, TAZ and NANOG signal form nuclear puncta (Fig. 7g and Supplementary Fig. 6f and g). Such nuclear puncta were not observed when TAZ or NANOG was knocked down (Fig. 7g and Supplementary Fig. 6f and g). Furthermore, proximity ligation assays confirmed the colocalization of TAZ with NANOG (Fig. 7h, i). Moreover, TAZ or NANOG knockout reduced tumorigenicity in serial transplantation models (Fig. 7j and Supplementary Fig. 6h). Collectively, these data suggested that targeting TAZ or NANOG inhibits cancer stemness and improves chemosensitivity in vivo.

## Discussion

CSCs play a key role in tumor progression^39^. However, the direct targeting of CSCs for therapeutic purposes is hardly feasible because specific markers to precisely identify CSCs are lacking in several malignancies^40^. Multiple stromal cells in the microenvironment were shown to regulate CSCs via biochemical factors^4,8,11–13^. Here, we advanced the understanding of the CSC niche by showing that biomechanical cues dictate the fate of CSCs by enhancing the transcriptional condensate formation of a pluripotency factor (Fig. 7k).

Although accumulating evidence has shown that matrix stiffness maintains CSCs properties, little is known about its underlying pathways^41^. Here, we uncovered a mechanism that TAZ forms phase-separated droplets with NANOG and consequently promotes NANOG’s ability to transcribe multiple pluripotency genes. Moreover, we revealed that N-terminal activation domain of NANOG is indispensable for the phase transition between NANOG and TAZ. Finally, we demonstrated in a mouse xenograft model that targeting TAZ or NANOG inhibits cancer stemness and improves chemosensitivity in vivo. Consistently, as reported, NANOG mediates the transcription of factors that regulate the differentiation decision of stem cells, including SOX2 and OCT4^42^. Furthermore, increasing evidence revealed that phase separation can concentrate signaling molecules at higher levels to accelerate gene transcription^37,43^. Similarly, recent studies on embryonic stem cells show that OCT4 can form phase-separated condensates with coactivator MED1 to activate pluripotency genes^37^. Taken together, others and our data indicate that the liquid–liquid phase separation of transcriptional factors play a crucial role in regulating both normal stem cells and CSCs.

As previously shown, mechanical cues can modulate proliferation and dormancy in other types of cancer cells in vitro or in mice^26,27^. Moreover, human breast cancers with poor histologic prognostic factors have higher stiffness values^28,44^. These observations raise the question of whether mechanoregulation is associated with breast cancer progression in patients^45^. Here, using clinical-practice UE for patients’ lesions and AFM for surgical samples, we provided clear evidence that matrix stiffness is associated with poor responses to NAC and worse long-term prognosis in patients with breast cancer. Our data unambiguously demonstrated that matrix stiffness is associated with CSC enrichment and is a clinically measurable biomarker to predict patient therapeutic efficacy and prognosis.

In summary, this study revealed a role of liquid–liquid phase separation as a signal nexus between niche stiffness and transcriptional activities of a pluripotency factor in CSCs. These findings extend the focus of CSC research and suggest that biomechanical cues are an attractive target of novel therapeutic strategies for CSCs.

## Methods

### Ethics statement

All procedures involving human samples were performed with the approval of the internal review and ethics boards of Sun Yat-Sen Memorial Hospital and all patients provided written informed consent in accordance with the Declaration of Helsinki. All mice used in this project were maintained under defined conditions at the Animal Experiment Center of Sun Yat-Sen University, and all animal experiments were approved by the Animal Care and Use Committee of Sun Yat-Sen University.

### Patients and human tumor samples

This study included a total of 563 female adult invasive breast cancer patients enrolled at the Sun Yat-Sen Memorial Hospital, Sun Yat-Sen University (Guangzhou, China) between 2009 and 2022. Among these cases, a cohort consisting of 124 invasive breast carcinoma patients (Supplementary Table 1) received NAC. In the NAC cohort, 114 and 10 patients had invasive ductal carcinoma and invasive lobular breast cancer, respectively. In addition, immunofluorescence staining for TAZ and NANOG was performed in 22 female patients and proximity ligation assay was performed in 29 cases who underwent neoadjuvant therapy at the Sun Yat-Sen Memorial between 2020 and 2022. Paired biopsy samples and surgically resected samples were collected from the same patients before and after neoadjuvant chemotherapy. The NAC regimens were as follows: doxorubicin 60 mg/m^2^ and cyclophosphamide 600 mg/m^2^ (AC) every 3 weeks for 4 cycles, followed by paclitaxel (80 mg/m^2^) weekly for 12 weeks, or docetaxel 75 mg/m^2^ and cyclophosphamide 600 mg/m^2^ (TC) every 3 weeks for 4 cycles. For HER2-positive patients, concomitant trastuzumab (initial loading and subsequent doses of 4 and 2 mg/kg/wk, respectively) was used weekly. Imaging examinations (breast ultrasound and magnetic resonance imaging) were applied to evaluate therapeutic effects according to the Response Evaluation Criteria in Solid Tumors (RECIST) guideline (version 1.1)^46^. CR was defined as disappearance of all lesions in both primary tumor and lymph nodes; PR was defined as at least a 30% reduction in the sum of the longest diameter of target lesions; PD was defined as at least a 20% increase in the sum of the longest diameter of target lesions; and SD was defined as neither sufficient shrinkage to qualify as PR nor sufficient increase to qualify as PD. Patients with CR or PR were classified as sensitive, and those with SD or PD were classified as resistant to neoadjuvant therapy.

Additionally, another cohort of 388 chemotherapy-naive invasive breast carcinoma samples (Supplementary Table 2) from Sun Yat-Sen Memorial Hospital, Sun Yat-Sen University (Guangzhou, China) between 2009 and 2022 were included for Kaplan-Meier survival analysis. The 388 breast cancer samples included invasive ductal carcinoma (*n* = 336), invasive lobular carcinoma (*n* = 32), mucinous carcinoma (*n* = 7), invasive micropapillary carcinoma (*n* = 6), medullary carcinoma (*n* = 3), metaplastic carcinoma (*n* = 2), and neuroendocrine tumor (*n* = 2).

### Animal studies

Six-week-old female wild-type NOD/SCID mice were purchased from the Animal Experiment Center of Sun Yat-Sen University. All mice were maintained at the Animal Experiment Center of Sun Yat-Sen University under the 14 h lights on/10 h lights off cycle and at 22 °C and 60% humidity. MCF-7 cells (1 × 10^6^ cells per mouse) or BT-474 cells (5 × 10^6^ cells per mouse) were mixed with hyaluronan-derived hydrogels and implanted into the mammary fat pads of 6-week-old female mice^38^. Specifically, tumor cells in 20 μL PBS suspension were embedded into 200 μL of soft or stiff formulations. After about 5 min gelation at room temperature, the cell-laden hydrogels were subcutaneously injected into fat pads of mice. Three days before inoculation of MCF-7 cells or BT-474 cells, each mouse was implanted with an estradiol pellet (1.7 mg, Innovative Research of America) to provide estrogen for tumor cells. Docetaxel 10 mg/kg i.p. once weekly was administered once the tumors were established. Tumor size was measured every week with a caliper, and the volume was calculated using the standard modified formula: Volume (mm^3^) = (length×height^2^)/2.

After 6 weeks of treatment, the xenografts were harvested for subsequent analysis. To detect CSCs proportion in xenografts, tumor tissues were dissociated mechanically and incubated with DMEM medium supplemented with 5% FBS, collagenase I (2 mg/ml, Worthington), and collagenase IV (2 mg/ml, Worthington) for 1 h at 37 °C followed by passaging through 40 μm filters as previously described^12^. The harvested tumor cells were used to measure CD44^high^CD24^low^ CSCs proportion.

Serial dilution and tumor formation assays were conducted as described previously^12^ with minor modifications. Briefly, 1 × 10^6^ MCF-7 or 5 × 10^6^ BT-474 cells without or with TAZ/NANOG knockout mixed with stiff hydrogels (9kPa) were injected into the mammary fat-pads of female 6-week-old NOD/SCID mice. The xenografts were harvested 6 weeks later, and tumors were dissociated mechanically and incubated with DMEM medium supplemented with 5% FBS, collagenase I (2 mg/ml, Worthington), and collagenase IV (2 mg/ml, Worthington) for 1 h at 37 °C followed by passaging through 40 μm filters and purified using CD326 microbeads (Miltenyi Biotec) according to the manufacturer’s instructions. Then, purified breast cancer cells were serially diluted in matrigel and transplanted into mammary fat-pads of a new batch of 6-weeks-old NOD/SCID mice. Tumor formation was monitored for 8 weeks following transplantation.

For animal experiments, survival endpoint was reached when tumors reached 1.5 cm diameter, or if animals appear distress or lost more than 20% of their body weight, as the Animal Care and Use Committee of Sun Yat-Sen University approved protocol. These limits were not exceeded in this study. At endpoint of animal experiments, mice were euthanized by intraperitoneal injection of pentobarbital sodium (150 mg/kg).

### Cell culture and polyacrylamide hydrogel preparation

MCF-7 cells (cat#HTB-22) and BT-474 cells (cat#HTB-20) cells were obtained from American Type Culture Collection (ATCC) and cultured according to standard protocols. All cell lines were maintained at 37 °C and 5% CO_2_ and tested for mycoplasma contamination. In some experiments, tumor cells were cultured on synthetic polyacrylamide substrate of high (9 kPa) or low (0.5 kPa) stiffness, as previously described^30^. Briefly, the sulfo-sanpah-activated hydrogels were coated with 10 μg/mL fibronectin (Sigma) and rinsed twice with PBS and DMEM before cell plating. AFM was applied to measure the stiffness of the hydrogel. Tumor cells were seeded at 5000–10,000 cells per cm^2^ on the above polyacrylamide-coated coverslips. After attachment, cells were maintained in DMEM containing 10% FBS and passaged when they grew to 90% confluence.

### Immunofluorescence and analysis

Formalin-fixed and paraffin-embedded surgical specimens or tumor tissues from animal experiments were sectioned at 4 μm for immunofluorescence staining. Antigen retrieval was performed using a pressure cooker for 3 min in antigen unmasking solution (citrate buffer, pH 6.0), followed by three washes in PBS and blocked with PBS containing 5% BSA for 15 min. Afterward, samples were incubated with mouse anti-human CK (1:200, clone: AE1/AE3, cat# ab27988, Abcam), goat anti-human ALDH1 (1:100, cat# AF5869, R&D Systems), rabbit anti-human TAZ (1:100, clone: E8E9G, cat# 83669, Cell Signaling Technology), goat anti-human OCT4 (1:100, cat# AF1759, R&D Systems), or rabbit anti-human SOX2 (1:100, clone: D6D9, cat# 3579, Cell Signaling Technology) antibodies overnight at 4 °C. Thereafter, slides were washed thrice with PBST, incubated with Alexa Fluor-conjugated secondary antibodies (1:300, cat# A-31570, cat# A-21202, cat# A-31571, cat# A-21432, cat# A-11055, cat# A-31572, or cat# A-21206, ThermoFisher Scientific) for 1 h at room temperature and stained with DAPI staining solution (Sigma). Images were obtained by laser scanning confocal microscopy (C2, Nikon).

For immunofluorescence staining of tumor cells cultured in vitro, MCF-7 and BT-474 cells were cultured on hydrogels for two weeks. Afterwards, cells were fixed with 4% paraformaldehyde for 15 min at RT, washed with PBS and blocked with 5% BSA in PBS for 1 h at RT. Then cells were incubated with rabbit anti-human TAZ (1:100, clone: E8E9G, cat# 83669, Cell Signaling Technology) overnight at 4 °C. After that, cells were washed with PBST and incubated with Alexa Fluor 488-conjugated donkey anti-rabbit IgG (1:300, cat# A-21206, ThermoFisher Scientific) for 1 h at RT and stained with DAPI. The laser scanning confocal microscopy (LSM980, Carl Zeiss) and ZEN 2012 software (Carl Zeiss) were used to acquire and analyse the images.

For immunofluorescence staining of TAZ and NANOG in human and murine tissue, freshly dissected tissue were fixed with 1% paraformaldehyde (in PBS) for 12 h at 4 °C and washed twice with PBS, followed by incubation in 15% sucrose for 2 h, and 30% sucrose overnight at 4 °C. Thereafter, the tissue was embedded in optimal cutting temperature (OCT) compound (Tissue-Tek) and frozen in dry ice. Frozen tissues were cut into 5 μm sections with cryotome (NX50, Thermo Fisher Scientific) and mounted on the gelatin-coated slides. The slides were blocked with 5% BSA (in PBS) for 15 min and incubated with goat anti-human ALDH1 (1:100, cat# AF5869, R&D Systems), rabbit anti-human TAZ (1:100, cat# 83669, Cell Signaling Technology) and mouse anti-human NANOG (1:100, clone: 1E6C4, cat# 4893, Cell Signaling Technology) antibodies overnight, followed by incubation of appropriate fluorescent secondary antibodies (1:300, cat# A-21447, cat# A-21206, or cat# A-31570, ThermoFisher Scientific) and DAPI. Images were taken with z-stack scanning by a Dragonfly Spinning Disc Confocal (Andor Technology) and maximal projection was processed with Imaris software (version 9.0, Bitplane). The signal colocalization of TAZ and NANOG was evaluated using the Imaris software as previously described^18^. Briefly, colocalization analysis was performed using an algorithm learning tool by rendering puncta with positive TAZ (green) and NANOG (red) signals, respectively, and building TAZ^+^NANOG^+^ puncta. Positive signal threshold was set by staining with negative control IgG. Then spot channels were generated for NANOG, TAZ and TAZ^+^ NANOG^+^ puncta, respectively. The colocalization level of NANOG with TAZ was calculated by dividing colocalizing spots of NANOG^+^TAZ^+^ by NANOG positive spots in the sample.

### Histology

Breast cancer tissues were fixed in formalin, embedded in paraffin, and sectioned for hematoxylin and eosin (H&E) staining. Hematoxylin and eosin (Solarbio) were applied according to the manufacturer’s instruction. For picrosirius red staining, we incubated 0.1% picrosirius red solution (Solarbio) with sections for 1 h at room temperature to stain collagen fiber in the tissues.

### TUNEL assay

For TUNEL assay, the slides were stained using the In Situ Cell Death Detection Kit (Roche). Tissue slices was incubated with the TUNEL reaction mixture at 37 °C for 30 min according to the manufacturer’s instructions, while the negative control was incubated with label solution (without terminal transferase). DAPI was then used for counterstaining of the nuclei, and images were obtained by laser scanning confocal microscopy (Nikon C2).

### AFM

AFM detection of clinical samples was performed as described before^47^. In brief, fresh tissue samples from patients were collected and directly snap frozen in liquid nitrogen. Frozen tissue blocks embedded in OCT compound were sectioned into 20 μm sections. Prior to AFM measurement, each section was immersed in PBS and thawed at room temperature. During the testing period, tissue samples were maintained in PBS containing protease inhibitor cocktail (Thermo) and propidium iodide (Sigma).

AFM indentations were performed using an Atomic Force Microscope (MFP 3D-BIO, Asylum Research). The silicon nitride cantilever (nominal spring constant k = 0.06 N/m) with a borosilicate glass spherical tip (tip radius 5.0 μm) was used for measurement. The cantilever was calibrated for sensitivity and spring constant before starting any measurement. Force-distance curves were plotted, and the Young’s modulus was calculated using Hertz model. The Poisson’s ratio was estimated to be 0.5 for soft biological materials. Data were analyzed using Igor Pro software (v 6.37, WaveMetrics). Ten regions per specimen were assessed and pooled to establish a mean Young’s modulus; statistical analysis was performed on the mean Young’s modulus.

### UE

UE^48^ was performed using a Hitachi HV-900 with a 5-13 MHz linear transducer (Hitachi Medical, Tokyo, Japan) before biopsy and breast cancer surgery. During the initial ultrasound survey of the tumor in the breast, the investigation depth was set so that the pectoralis muscle was visualized along the posterior margin of the field of view. Initial gain settings were adjusted so that fat at all levels was displayed as a midlevel grey and solid mass was hypoechoic, while the skin was hyperechoic. Images were monitored in real-time and displayed in split-screen mode with the greyscale ultrasound B-mode and UE images on the right and left, respectively. A square region of interest (ROI), which includes subcutaneous fat at the top of the breast, pectoral muscle at the bottom of the breast, and >5 mm of breast parenchyma adjacent to the targeted lesion, was set for elastography acquisition. The probe was placed gently on the breast surface to obtain elasticity images until the pressure bar indicated a stable 3-4 index. The UE images were illustrated with 256 color mapping for each pixel based on the extent of strain. The color scale ranged from red (indicating soft) to blue (hard elasticity). To decide each lesion ultrasound elastic score, all images were retrospectively analyzed by two radiologists, who had at least 3 years’ experience in breast UE. All lesions were scored according to a 5-point scoring system proposed by Itoh et al^48^. Ultrasound B-mode images were co-registered with the elastography color maps to guide tumor segmentation. Breast radiologists segmented tumor outlines, set as ROI A, based on the hypoechoic solid masses in B-mode images. Then a corresponding ROI B was selected from adjacent breast tissue of the same depth as an internal reference. The strain ratio was calculated as the average strain of ROI B/ ROI A ratio, which reflected the stiffness property of the lesion^49^.

### Apoptosis analysis

MCF-7 and BT-474 cells cultured on hydrogels for 2 weeks were treated with chemotherapeutic agents (docetaxel or cisplatin) for 24 h. Then cells were dissociated by 0.25% trypsin-EDTA and harvested by centrifugation 1000 rpm for 5 min. To detect apoptotic cells, MCF-7 or BT-474 cells (1× 10^5^ per sample) were incubated with 100 μL binding buffer (Biolegend) containing 5 μL APC-conjugated Annexin V (1:20, cat# 640920, Biolegend) for 15 min at room temperature. After incubation, the cells were washed and resuspended in binding buffer (200 μL) containing 5 μL propidium iodide (PI, Biolegend) staining solution and detected in a flow cytometry immediately. Data were analyzed by FlowJo (Version 10, Treestar).

### Flow cytometry

For cell surface marker staining and flow cytometric analysis, MCF-7 or BT-474 cells were resuspended in PBS containing 1% FBS and incubated with PE anti-human CD24 antibody (1:20, clone: ML5, Cat# 311106, BioLegend) and APC anti-human CD44 antibody (1:20, clone: ML5BJ18, Cat# 338806, BioLegend) for 30 min at 4 °C. Afterwards, cells were washed with PBS. ALDH1 activity was measured using the ALDEFLUOR kit (Cat# 01700, Stem Cell Technologies) according to the manufacturer’s instructions. Specimens were subsequently analyzed by a CytoFlex Flow cytometer (Beckman Coulter). Data were analyzed using FlowJo software (version 10, Treestar).

### Cell counting kit-8 (CCK8) assay

CCK8 assay was used to determine cell viability. Briefly, 1000 cells retrieved from hydrogels were seeded onto 96-well plates and incubated overnight at 37 °C. The cells were treated with the indicated chemotherapeutic agents for 24 h. Thereafter, CCK8 solution (Dojindo Laboratories) was added to the cells and incubated for 2 h at 37 °C. The absorbance was measured with a microplate reader (infinite M200 PRO) at 450 nm. Six replicate wells were included in each analysis.

### Western blot

Proteins were extracted from the cells using RIPA buffer, resolved by SDS-polyacrylamide gels, and then transferred to poly-vinylidene difluoride (PVDF) membranes. The membranes were probed with rabbit anti-human TAZ (1:1000, cat# 83669), rabbit anti-human total caspase-3 (1:1000, cat# 9662), rabbit anti-human cleaved caspase-3 (1:1000, cat# 9661), rabbit anti-human total PARP (1:1000, clone: 46D11, cat# 9532), rabbit anti-human cleaved PARP (1:1000, clone: D64E10, cat# 5625), mouse anti-human NANOG (1:1000, cat# 4893), or rabbit anti-human SOX2 (1:1000, cat# 3579) from Cell Signaling Technology, or rabbit anti-human OCT4 (1:1000, abcam, cat# ab19857), rabbit anti-human NANOG (1:1000, clone: EPR2027(2), cat# ab109250, Abcam), mouse anti-human GAPDH (1:10000, clone: 1E6D9, cat# 60004-1-Ig, Proteintech), or rabbit anti-human α-tubulin (1:10000, cat# 11224-1-AP, Proteintech) antibodies overnight at 4 °C. HRP-linked anti-rabbit IgG (1:3000, cat# 7074, Cell Signaling Technology) or anti-mouse IgG (1:3000, cat# 7076, Cell Signaling Technology) was used and the antigen-antibody reaction was visualized by an enhanced chemiluminescence assay (ECL, Thermo). Densitometry was performed using ImageJ software. GAPDH or α-tubulin run on the same blot were used as the loading controls unless otherwise indicated.

### Protein expression and purification

N-terminal His6-tagged full-length human TAZ, full-length human NANOG, human NANOG-CAD and human NANOG-NAD plasmids were synthesized by chemical synthesis method and inserted into the NdeI/XhoI sites of pET-28a (+) plasmids and confirmed by DNA sequencing analysis. *E. coli* (DE3) were grown at 37 °C and induced with 0.5 mM isopropyl β-D-1-thiogalactopyranoside (IPTG) for 16 h at 20 °C. Cells were pelleted, resuspended, and lysed in the xTractor buffer (Cat#635623; Takara) supplemented with an EDTA-free protease inhibitor cocktail (Cat#87786, Thermo) by rocking at room temperature for 20 min. After centrifugation at 16,000 g for 30 min at 4 °C, the supernatant was collected. The soluble protein was purified using pre-balanced nickel columns (Cat#635623; Takara) and eluted with the following buffer: 20 mM Na_3_PO_4_, 500 mM NaCl, 500 mM imidazole, and pH 7.6. Amicon Ultra-0.5 spin columns (Cat#Z677094; Merck-Millipore) were used for further concentration and buffer exchange. Purified protein was quantified using a ND-2000C NanoDrop spectrophotometer (NanoDrop Technologies) with OD 280 nm and verified by Coomassie staining. Recombinant protein was diluted in storage buffer (50 mM Tris-HCl, 500 mM KCl, 1 mM DTT, and 5% glycerol), flash-frozen in liquid nitrogen, and stored at -80 °C.

### In vitro phase separation assay

Recombinant protein diluted in buffer at physiological salt conditions (25 mM HEPES pH 7.5, 150 mM NaCl, and 1 mM DTT) was mixed at a 1:1 stoichiometry at indicated final concentrations at 37 °C. The protein mixtures in a 20 μL solution were added into a non-binding 24-well plate (Corning) coated with 20 mg/ml BSA. The images or movies were taken after 15-20 min incubation right after all the condensates had settled to the bottom of the plate using laser scanning confocal microscopy (LSM880, Carl Zeiss).

### In vivo phase separation experiments

HEK293T cells (cat# CRL-3216) were obtained from the American Type Culture Collection. HEK293T cells were transfected with indicated plasmids and cultured for 48 h at 37 °C. Confocal images were taken by laser scanning confocal microscopy (LSM880, Carl Zeiss) equipped with a 63 × oil immersion objective (Plan-Apochromat 63x/1.4 Oil DIC M27). Excitation/emission wavelengths were 488 nm/503-549 nm for EGFP-labeled TAZ samples, and 561 nm/602-632 nm for mCherry-labeled NANOG samples.

### Fluorescence recovery after photo-bleaching assay (FRAP)

HEK293T cells were transfected with indicated plasmids, and cultured for 48 h at 37 °C. The FRAP assay was performed using a confocal microscope (LSM880, Carl Zeiss). The time-lapse images were taken at a rate of 50 ms/frame for 30 s. The condensates were bleached with 488 nm or 561 nm laser lines after 2 scans. The time right after photobleaching was set to time 0 s. At each time point, the fluorescence intensities of photobleached areas from individual images were measured by ImageJ and normalized against the pre-bleached fluorescence intensity times. The normalized intensities were plotted to determine the percentage of bleached fluorescence recovery using GraphPad Prism 9.

### Sedimentation assay

Sedimentation assay was performed as previously reported^50^. For cell lysis, 1 × 10^6^ cells were flash frozen with liquid nitrogen and stored at -80 °C. Then the cell pellet was resuspended in lysis buffer (150 mM HEPES pH 7.5, 150 mM NaCl, 1 mM EDTA, 0.2% NP-40, 2× protease inhibitor cocktail, 1 mM PMSF, and 1 mM DTT). Homogenization was performed using glass beads beating (30 s of strokes/ 30 s of cooling on ice) for four times. Then the samples were clarified by centrifugation at 650 × g for 2 min at 4 °C. Then half of the supernatant was collected and incubated at 4 °C for 1 h before centrifugation at 100, 000 × g to separate soluble and pellet fractions. Then the supernatant, soluble, and pellet fractions at equal volumes were subjected to SDS-PAGE followed by Western blotting analysis.

### RNA extraction, reverse transcription, and real-time quantitative reverse transcription polymerase chain reaction (RT-qPCR)

Total RNA was extracted using Trizol reagent (Invitrogen) according to the manufacturer’s protocol. RNA (500 ng) was reverse-transcribed using PrimeScript™ RT Master Mix (Cat#RR820A, Takara). The resulting cDNA was analyzed using RT–qPCR by TB Green® Premix Ex Taq™ II (Cat#RR036A, Takara) and LightCycler 480 II system (Roche). β-actin was used as control. The following primers were used:

*Sox2* forward: TACAGCATGTCCTACTCGCAG;

*Sox2* reverse: GAGGAAGAGGTAACCACAGGG;

*Pou5f1* (encoding OCT4) forward: CTGGGTTGATCCTCGGACCT;

*Pou5f1* (encoding OCT4) reverse: CCATCGGAGTTGCTCTCCA;

*Fgf4* forward: CTCGCCCTTCTTCACCGATG;

*Fgf4* reverse: GTAGGACTCGTAGGCGTTGTA;

*β-actin* forward: GCCGACAGGATGCAGAAGGAGATCA;

*β-actin* reverse: AAGCATTTGCGGTGGACGATGGA.

### Immunofluorescence with RNA FISH

HEK293T cells transfected with indicated plasmids were plated on coverslip and incubated at 37 °C for 48 h. Then cells were fixed in 4% paraformaldehyde for 20 min, washed three times in PBS and permeabilized with 0.5% v/v Triton (Cat#T8787, Sigma). After prehybridization at 50 °C for 2 h, 25 nM encoding probes in encoding hybridization buffer was added to the cell-containing coverslip. Samples were incubated in a hybridization oven at 50 °C overnight. Cells were washed with 2× saline-sodium citrate buffer (SSC) twice at 50 °C for 5 min, and incubated with 2×SSC and 50% v/v formamide three times at 50 °C for 25 min. Then samples were washed four times with PBST (0.1% Tween in PBS), once with PBS at room temperature for 5 min, blocked with normal goat serum for 1 h. The samples were incubated with anti-digoxin-fluorescein antibody (1:100, cat# 11207741910, Roche) and mouse anti-human TAZ antibody (1:100, clone: CL0371, cat# ab242313, Abcam) at 4 °C overnight. After that, samples were washed thrice with PBST, incubated with Alexa Fluor 555-conjugated donkey anti-mouse IgG (1:300, cat# A-31570, ThermoFisher Scientific) for 1 h at room temperature and stained with DAPI staining solution (Sigma). Images were taken by laser scanning confocal microscopy (LSM 880, Carl Zeiss).

### Lentiviral vector-mediated silencing or overexpression

Lentivirus packaging was supplied by the Guangzhou IGE Biotechnology. In some experiments, human *Taz* (NM_001168278.3), *Nanog* (NM_024865.4), *Sox2* (NM_003106.4), and *Pou5f1* (NM_002701.6) gene were cloned into lentiviral overexpression vectors pCDH-CMV-MCS-EF1-neo and transduced MCF-7 and BT-474 cells. The lentiviral vector plasmids, pLKO.1-hygro (Addgene), were used to clone the following sense sequences to construct the stable clones:

shTAZ-1: GCGTTCTTGTGACAGATTATA;

shTAZ-2: GCGATGAATCAGCCTCTGAAT;

shNANOG: AAGGGTTAAGCTGTAACATAC;

shGFP (negative control): GCAAGCTGACCCTGAAGTTCAT.

We generated stable knockdown cells as previously described^51,52^. Briefly, lentiviral particles (multiplicity of infection: 30) were used to infect MCF-7 and BT-474 cells with 10 μg/mL polybrene (Sigma-Aldrich) at 37 °C overnight. Stable pools were selected with 800 μg/mL G418 for 7 days or 50 μg/mL hygromycin B for 5 days. Knockdown and overexpression efficiency of proteins was evaluated by Western blotting.

### CRISPR-mediated gene knockout

The sequences targeting TAZ or NANOG were cloned into lentivirus vector LentiCRISPR v2-Puro for gene knockout. The guide sequence for sgRNA used were TAZ gRNA: 5’-TGTCTAGGTCCTGCGTGACG-3’ and NANOG gRNA: 5’-CAGTCGGATGCTTCAAAGCA-3’^53,54^. MCF-7 and BT-474 tumor cells (2 × 10^5^ cells per well) in 6-well plates were transfected with lentiviral particles (multiplicity of infection (MOI) of 5) and 5 μg/mL Polybrene (Sigma-Aldrich) for 12 hr. The transduced cells were selected with 2.5 μg/ml puromycin for 2 weeks to obtain the TAZ/NANOG knockout tumor cells. For gain and loss function experiments, lentiviral plasmid expressing TAZ, NANOG, SOX2 or OCT4 were transfected into TAZ/NANOG knockout cells and selected in 800 μg/ml G418 for 7 days.

### Chromatin-immunoprecipitation-quantitative polymerase chain reaction (ChIP-qPCR) assay

CHIP assay was carried out using the ChIP Assay Kit (Merck Millipore) according to the kit’s instruction. In brief, MCF-7 cells were cultured on soft or stiff hydrogels for 2 weeks. MCF-7 cells (5 × 10^6^) were crosslinked with 1% formaldehyde for 10 min at room temperature. Then samples were lysed and sonicated to shear chromatin DNA with an Ultrasonic Processor (Sonics VCX130) for 10 cycles (50% amplitude, 10 s on and 20 s off). The chromatin was incubated with TAZ antibody (1:50, clone: E9J5A, cat# 72804, Cell Signaling Technology), NANOG antibody (1:25, cat# 3580, Cell Signaling Technology), or IgG overnight at 4 °C. Then the captured chromatin was eluted, crosslinks reverted. The DNA sample was subjected to PCR analysis using the following primers:

*Sox2* promoter forward: 5′- GCGTCCCATCCTCATTTAAG -3′

*Sox2* promoter reverse: 5′- AGCAACAGGTCACACCACAC -3′

*Pou5f1* promoter forward: 5′- TTGGGGAGCAGGAAGCAGTC -3′

*Pou5f1* promoter reverse: 5′- GACAATGGCCTTGGCTGGAC -3′

### Preparation of hyaluronan-derived hydrogels

HyStem-C kit (Advanced Biomatrix) was applied to prepare hydrogels of defined stiffness (soft: 0.5 kPa and stiff: 9 kPa) for subcutaneous injection in animal experiments^35,38^. Stock concentrations of 10 mg/mL Glycosil, 10 mg/mL Gelin-S, and 5 mg/mL Extralink were prepared to obtain soft hydrogels and 2 × Glycosil and 5 × Extralink for stiff hydrogels. Solutions were mixed at 1:5 ratios of Extralink/ (Glycosil + Gelin) for soft hydrogels and 5 × Extralink/ (2 × Glycosil + Gelin) for stiff hydrogels. AFM was applied to measure the stiffness of the hydrogel.

### Positron emission tomography/computed tomography (PET/CT) imaging

The therapeutic effect on mice was assessed by ^18^F-fluorodeoxyglucose (^18^F-FDG) PET/CT after six weeks of treatment. Before PET/CT scanning, mice were fasted for 8 h, anesthetized with 40 mg/kg pentobarbital sodium, and injected with 5 uci/g ^18^F-FDG in 100 μL saline via tail vein. A 15-min static scan was performed 40 min after ^18^F-FDG injection with an Inveon micro-PET/CT Scanner (Siemens, Germany). The micro-PET images were corrected for attenuation, scatter, normalization, and camera dead time, and co-registered with micro-CT images. The tumor uptake of ^18^F-FDG was calculated as the standardized uptake value (SUV) in three-dimensional ROIs.

### Proximity ligation assay (PLA)

PLA was performed using the Duolink in situ PLA Detection Kit (Sigma). Briefly, human breast cancer tissue or xenograft sections were dewaxed, hydrated and antigen repaired. After washed in PBS for 3 times, sections were incubated with Duolink blocking solution for 60 min at 37 °C. Primary mouse anti-human NANOG (1:100, cat# 4893, Cell Signaling Technology) and rabbit anti-human TAZ (1:100, cat# 83669, Cell Signaling Technology) antibodies in blocking solution was incubated for overnight at 4 °C. The PLA Probe incubation, ligation, and amplification reactions were performed according to the manufacturer’s instructions. Then, tissues were washed in Wash Buffer B and incubated with goat anti-human CK antibody (1:200, cat# ab219271, Abcam) for 1 h at RT, followed by incubated with Alexa Fluor 488-conjugated donkey anti-goat IgG (1:300, cat# A-11055, Thermo Fisher Scientific) for 1 h at RT. Finally, sections were mounted in Duolink in situ mounting medium with DAPI. Images were taken as z-stacks with a 0.25 μm step size by a Dragonfly Spinning Disc Confocal (Andor Technology). Max projections of z-stack were processed and analyzed by Imaris software.

### Statistical analysis

Statistical analyses were performed using GraphPad Prism 9 (GraphPad Software, La Jolla, CA) or SPSS (version 23, IBM Analytics). Data are shown as mean ± standard deviation (SD) or mean ± standard error of mean (SEM). Kaplan-Meier survival curves were plotted, and the log-rank (Mantel-Cox) test was used to compare survival curves. X-tile statistical software (v3.6.1) was used to group clinical samples and to determine an optimal cutoff point by a minimal p-value approach. For analysis of statistical difference between two groups, a two-tailed Mann-Whitney U test or two-tailed Student’s t test with 95% confidence interval was applied. Two-sided one-way or two-way ANOVA with Tukey test was applied for the statistical analysis of three or more groups. Significance was defined as p < 0.05. All experiments were replicated at least 3 times. The exact number of independent experiments and the statistical details are shown in the figure legends.

### Reporting summary

Further information on research design is available in the Nature Portfolio Reporting Summary linked to this article.

## Supplementary information

Supplementary Information

Reporting Summary

## Data Availability

All the data supporting the findings of this study are available within the main text of this article and its Supplementary Information. Source data are provided with this paper.

## Source data

Source Data
