# Supplementary material for: Niche stiffness sustains cancer stemness via TAZ and NANOG phase separation
Source: Nat Commun. 2023 Jan 16;14:238. doi: 10.1038/s41467-023-35856-y (PMC9842735; doi:10.1038/s41467-023-35856-y)
Supplement: Supplementary file 1 — Supplementary Information [file 41467_2023_35856_MOESM1_ESM.pdf]

Supplementary Information

Niche stiffness sustains cancer stemness via TAZ and NANOG phase separation

Authors: Xinwei Liu; Yingying Ye; Liling Zhu; Xiaoyun Xiao; Boxuan Zhou; Yuanting Gu; Hang Si; Huixin Liang; Mingzhu Liu; Jiaqian Li; Qiongchao Jiang; Jiang Li; Shubin Yu; Ruiying Ma; Shicheng Su; Jian-You Liao; Qiyi Zhao

Supplementary Figures 1-6

Supplementary Tables 1, 2

Supplementary Figures

Supplementary Figure 1.

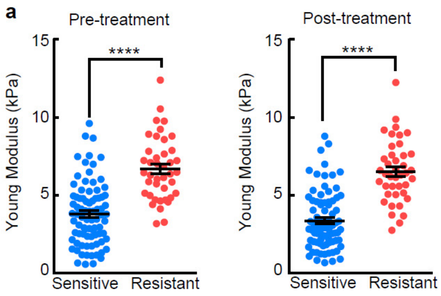

Supplementary Fig. 1. Matrix stiffness is correlated with chemoresistance and poor survival in patients with breast cancer. Related to Fig. 1.

a Quantitation of tumor Young Modulus (kPa) in Fig.1a. Sensitive group, n = 83 patients; Resistant group, n = 41 patients. Mean ± SEM, ****p < 0.0001, by two-sided Student's t test.

Supplementary Figure 2.

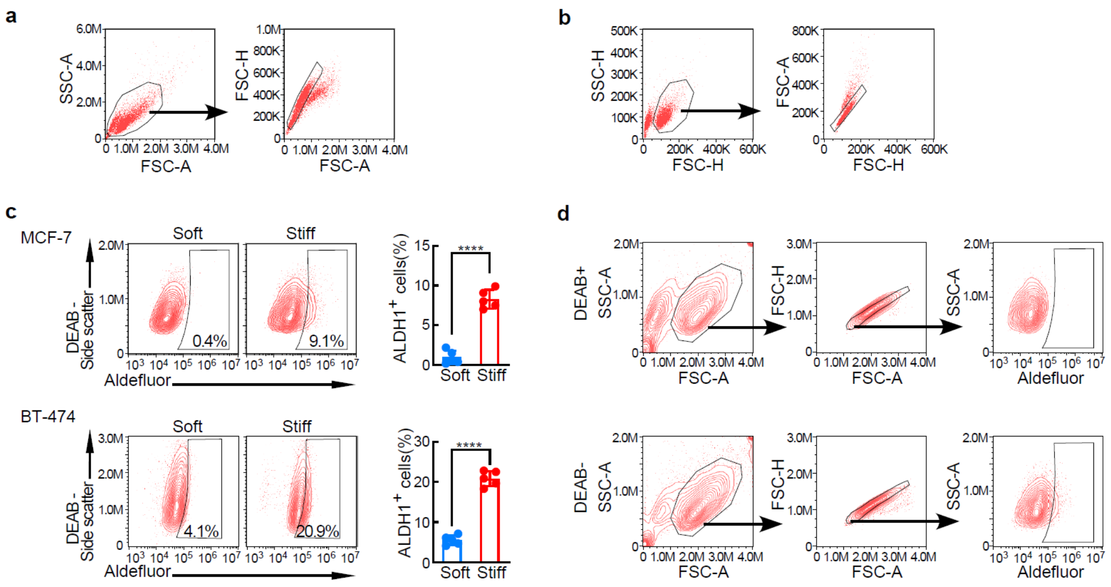

Supplementary Fig. 2. Matrix stiffness induces chemoresistance and enriches CSCs in breast cancer cells. Related to Fig. 3.

MCF-7 and BT-474 cells were cultured on soft (0.5 kPa) or stiff (9 kPa) supports for 2 weeks before subsequent analysis.

a Gating strategy for fig. 3a.

b Gating strategy for fig. 3e.

c The percentage of $ALDH1^+$ cells in MCF-7 and BT-474 cells cultured on soft or stiff supports determined by flow cytometry. Representative image (left) and qualification (right) are shown. Data from five biologically independent experiments; mean ± SD, ****$p < 0.0001$, by two-sided Student's t test.

d Gating strategy for supplementary fig. 2c. Breast cancer cells were incubated with Aldefluor in presence or absence of DEAB, a competitive inhibitor of ALDH1. The

sorting gate of the $ALDH1^+$ CSCs was established using DEAB-treated cells as a reference.

Supplementary Figure 3.

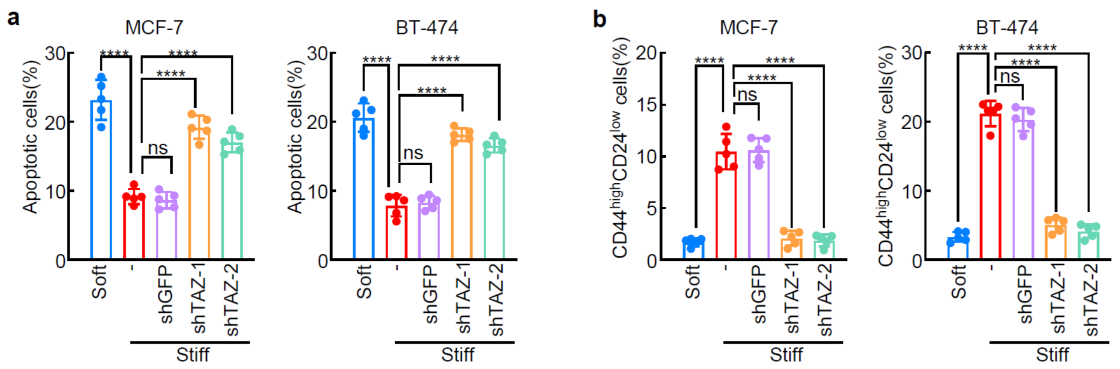

Supplementary Fig. 3. TAZ regulates chemoresistance and cancer stemness *in vitro*. Related to Fig. 4.

MCF-7 and BT-474 cells were cultured on soft or stiff supports for 2 weeks. In some experiments, cells were pre-transduced with TAZ shRNA (shTAZ-1 or shTAZ-2) or GFP shRNA (shGFP).

a Quantitation of apoptotic cells (Annexin V^+) percentage in Fig. 4d (n = 5 biologically independent experiments). Mean ± SD, ****p < 0.0001; for MCF-7 cells, ns = 0.9886; for BT-474 cells, ns = 0.9873; by two-sided one-way ANOVA with Tukey test.

b Quantitation of $CD44^{high}CD24^{low}$ cancer cells percentage in Fig. 4f (n = 5 biologically independent experiments). Mean ± SD, ****p < 0.0001; for MCF-7 cells, ns = 0.9988; for BT-474 cells, ns = 0.8381; by two-sided one-way ANOVA with Tukey test.

Supplementary Figure 4.

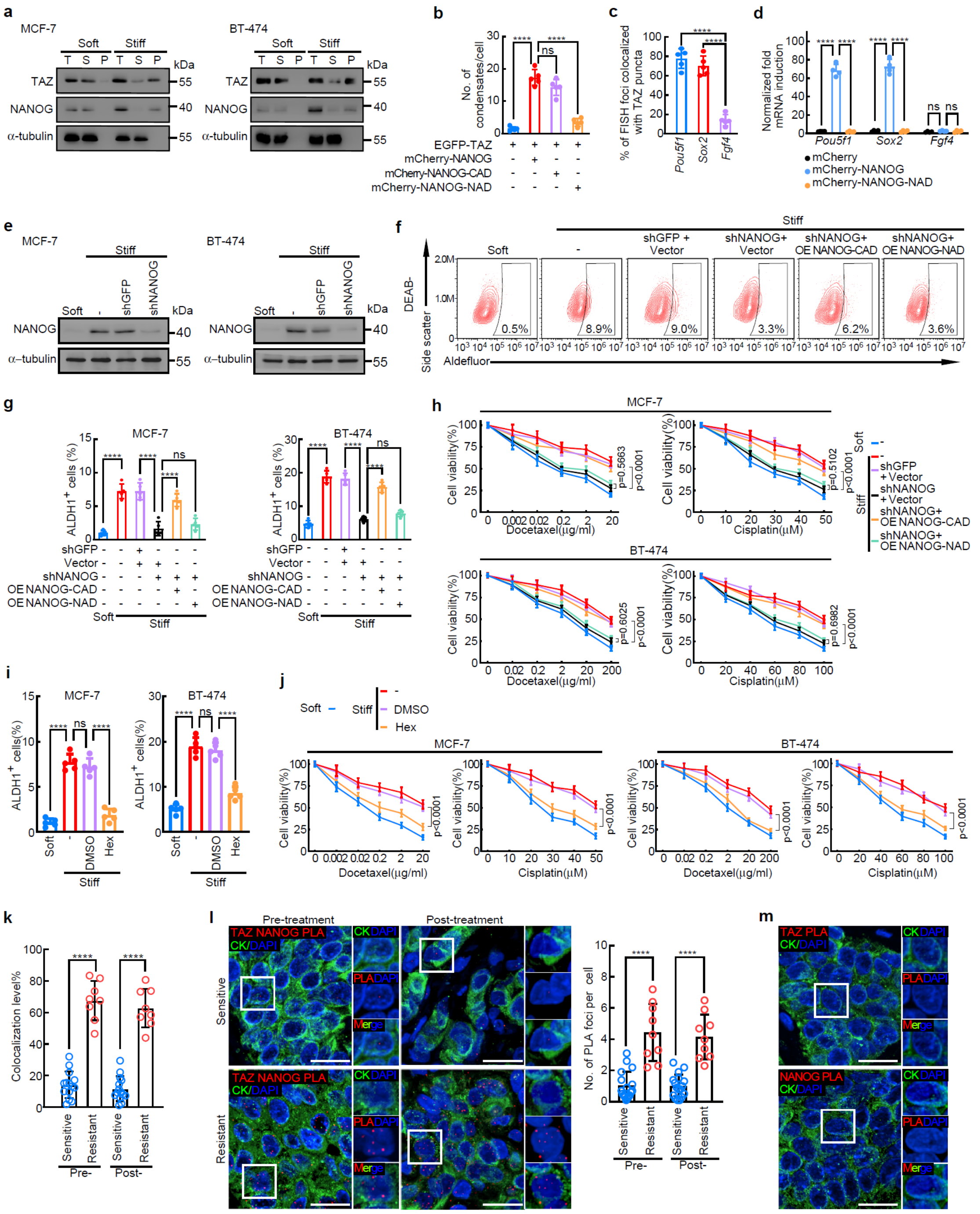

Supplementary Fig. 4. TAZ regulates SOX2 and OCT4 expression through phase separation with NANOG. Related to Fig. 5.

a The amount of TAZ and NANOG in pellet versus soluble fraction increases in MCF-7 and BT-474 cells cultured on stiff hydrogels, compared with cells on soft hydrogels.

T, lysates of total protein sample. Total lysates were centrifuged for separating soluble (S) and pellet (P) fractions. Blot shown is representative of results from three experiments. For data presented in the same figure panel, the samples were derived from the same experiment and blots were processed in parallel.

b Quantification of the number of condensates in HEK293T cells that showed nuclear puncta in Fig.5g. 10 cells were assessed per group; experiments were independently repeated 5 times. Mean ± SD; ns= 0.0908; ****p<0.0001 by two-sided one-way ANOVA with Tukey test.

c Quantification of RNA-FISH foci colocalized with TAZ puncta in the nucleus of HEK293T cells in Fig.5h. 10 cells were assessed per group; experiments were independently repeated 5 times. Mean ± SD; ****p<0.0001 by two-sided one-way ANOVA with Tukey test.

d Quantitative PCR with reverse transcription (RT-qPCR) analysis of *Pou5f1*, *Sox2* and *Fgf4* mRNA expression in HEK293T cells transfected with TAZ (WT) and indicated NANOG mutants (n=4 biologically independent experiments). Mean ± SD; ****p<0.0001; ns = 0.9222 (mCherry) and 0.9847 (mCherry-NANOG-NAD); by two-sided one-way ANOVA with Tukey test.

e-h NANOG was knockdown with shRNA in MCF-7 and BT-474 cells. NANOG-CAD mutant, NANOG-NAD mutant or vector was overexpressed in NANOG silenced tumor cells. Tumor cells were cultured on hydrogels for 2 weeks.

e The knockdown efficiency of NANOG was evaluated by western blotting. This blot is representative of three independent experiments.

f The proportion of $ALDH1^+$ tumor cells was measured by flow cytometry. Representative flow cytometry plots of MCF-7 cells are shown.

g Quantifications of the proportion of $ALDH1^+$ tumor cells in MCF-7 and BT-474 cells measured by flow cytometry as **(f)** (n = 5 biologically independent experiments). Mean ± SD, ****p<0.0001; for MCF-7 cells, ns = 0.8939; for BT-474 cells, ns =0.4083, by two-sided one-way ANOVA with Tukey test.

h Cell viability was determined by CCK8 assay. n = 4 biologically independent experiments.

i, j MCF-7 and BT-474 cells were cultured on hydrogels for 2 weeks and treated with DMSO or 6% 1,6-hexanediol (Hex) in cell culture media for 1 minute at 37 °C once a day.

i The proportion of $ALDH1^+$ tumor cells was measured by flow cytometry (n=5 biologically independent experiments). Mean ± SD, ****p<0.0001; for MCF-7 cells, ns = 0.7409; for BT-474 cells, ns =0.8496, by two-sided one-way ANOVA with Tukey test.

j Cell viability was determined by CCK8 assay. n = 4 biologically independent samples per group.

h-j Mean ± SD, ****p ＜ 0.0001; ns= no significance, by two-sided one-way ANOVA with Tukey test.

k The colocalization levels of NANOG with TAZ puncta in cell nucleus in Fig.5i were evaluated by Imaris software. 10 fields were assessed per patient. 14 patients achieved PR in sensitive group; 7 patients achieved SD and 1 patient achieved PD in resistant group; mean ± SD, ****$p < 0.0001$ by two-sided student's t test. Pre-, pre-treatment; post-, post-treatment.

l Representative images (maximal projections in z) and qualification of TAZ and NANOG antibodies for PLA in paired samples of breast patients before and after NAC. Scale bar, 20 μm. Sensitive group, n = 17 patients; resistant group, n = 9 patients; mean ± SD, ****$p < 0.0001$ by two-sided student's t test.

m Tissue sections of breast patients received NAC incubated with single antibody (TAZ or NANOG) were served as controls of Supplementary Fig. 4l. Representative images of three independent experiments are shown. White box delineates the insets of higher magnification views. Scale bar, 20 μm.

Supplementary Figure 5.

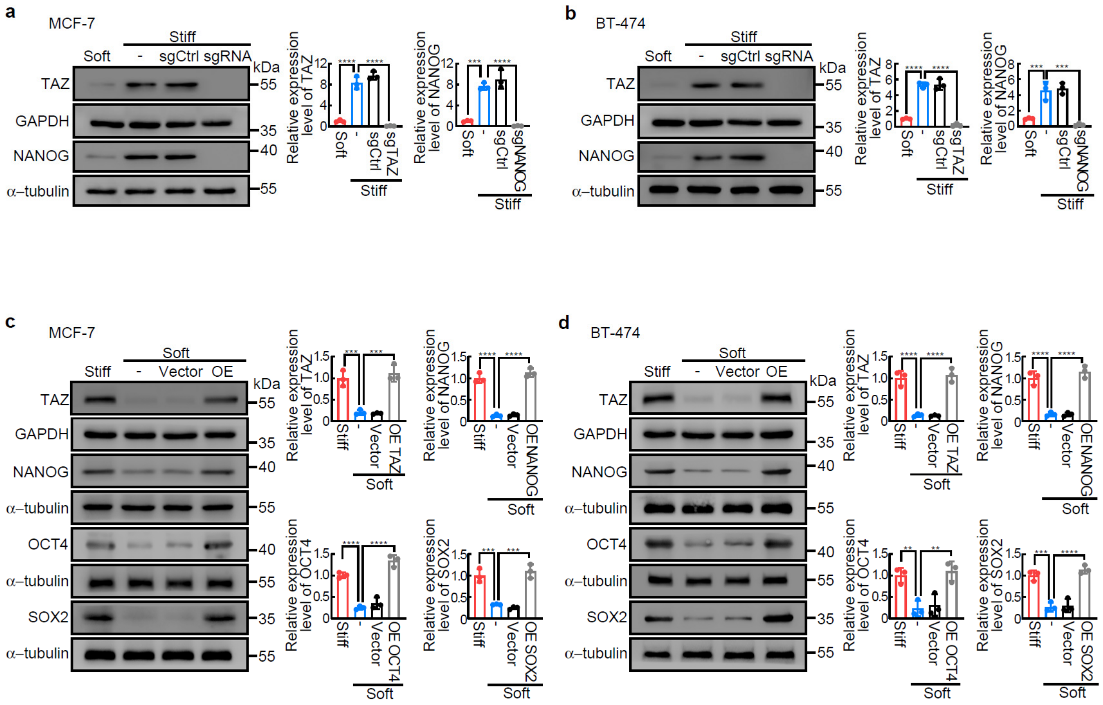

Supplementary Fig. 5. Roles of TAZ and NANOG on SOX2 and OCT4. Related to Fig. 6.

a, b MCF-7 **(a)** and BT-474 cells **(b)** were transduced without or with lentivirus carrying control sgRNAs or sgRNAs targeting TAZ or NANOG and cultured on soft or stiff hydrogels. Knockout efficiency was verified via western blotting (n = 3 biologically independent experiments). Mean ± SD, ****p<0.0001; for MCF-7 cells, ***p=0.0001; for BT-474 cells, ***p=0.0009 (soft) and 0.0002 (sgNANOG); by two-sided one-way ANOVA with Tukey test.

c, d MCF-7 **(c)** and BT-474 cells **(d)** were transduced without or with lentivirus carrying an empty vector or vector expressing TAZ, NANOG, SOX2 or OCT4. Overexpression efficiency was verified via western blotting (n = 3 biologically independent experiments). OE, overexpression. Mean ± SD, ****p<0.0001; for TAZ expression in

MCF-7 cells, ***p=0.0005 (stiff) and 0.0002 (OE TAZ); for SOX2 expression in MCF-7 cells, ***p=0.0004 (stiff) and 0.0002 (OE SOX2); for OCT4 expression in BT-474 cells, **p=0.0092 (stiff) and 0.0046(OE OCT4); for SOX2 expression in BT-474 cells, ***p=0.0003 (stiff); by two-sided one-way ANOVA with Tukey test.

Supplementary Figure 6.

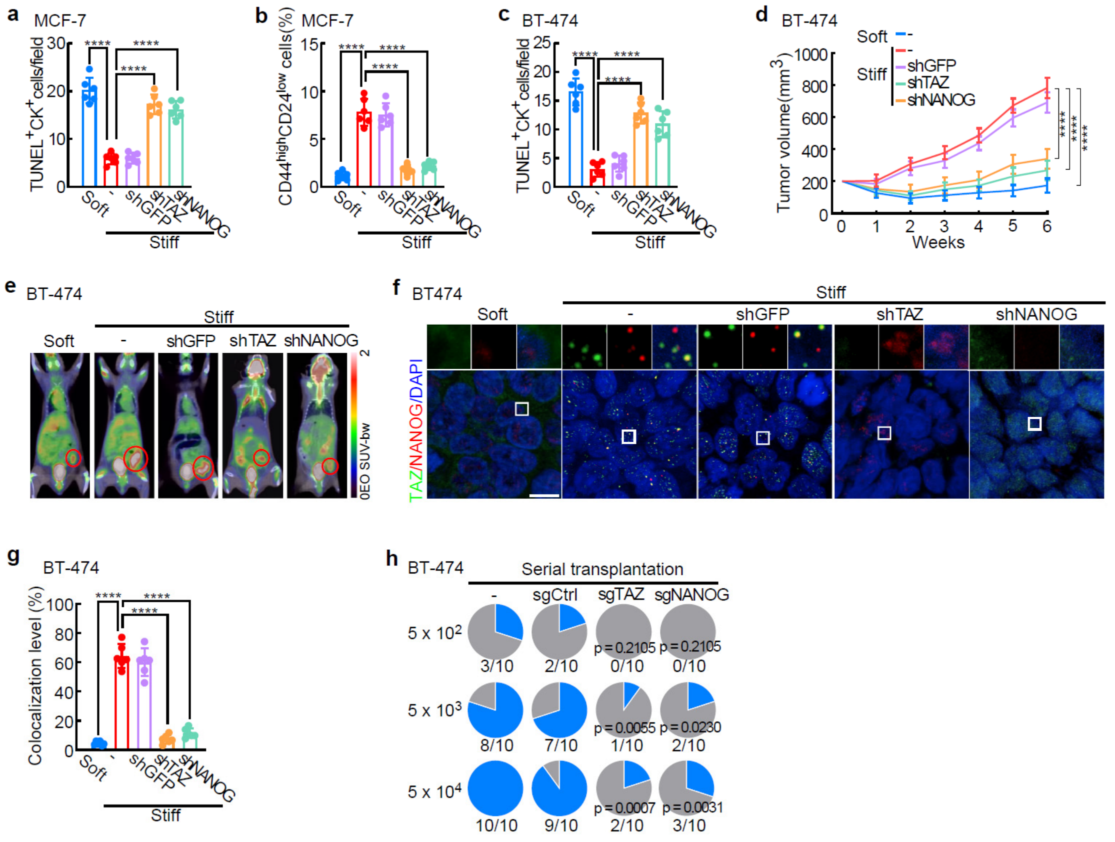

Supplementary Fig. 6. TAZ and NANOG regulate cancer stemness, and chemoresistance *in vivo.* Related to Fig. 7.

a-b MCF-7 cells transduced with shGFP, shTAZ or shNANOG were mixed with hyaluronan-derived hydrogels and injected into the mammary fat pads of NOD/SCID mice. Mice were treated with docetaxel (Doc) per week (n = 6 biologically independent animals per group).

a Quantitation of TUNEL$^+$ CK$^+$ cells in Fig.7a.

b Quantitation of CD44^{high}CD24^{low} cells percentage in Fig.7d.

c-g BT-474 cells transduced without or with shGFP, shTAZ or shNANOG were mixed with hyaluronan-derived hydrogels and injected into the mammary fat pads of

NOD/SCID mice. Mice were treated with docetaxel (Doc) weekly (n = 6 biologically independent mice per group).

c, Quantifications of TUNEL$^+$ CK$^+$ tumor cells in the BT-474 xenografts.

d, Tumor growth curves of BT-474 xenografts are shown.

e, Representative images of BT-474 xenografts monitored by PET-CT at the sixth week.

f, Representative images (maximal projections in z) of TAZ and NANOG immunofluorescent staining in the xenografts of BT-474. White box delineates the insets of higher magnification views. Scale bars, 10 μm.

g, The colocalization levels of NANOG with TAZ as shown in **(f)** were evaluated by Imaris software, 5 fields per tumor from 6 mice per group were assessed.

a-d, g, Mean ± SD, ****p < 0.0001, by two-sided one-way ANOVA with Tukey test.

h, Tumorigenesis incidences of the secondary tumor in serial transplantation models of BT-474 cells are shown (n=10 biologically independent animals per group). P values compared with the first group by two-sided Fisher's exact test were shown as indicated.

Supplementary Tables

Supplementary Table 1. Clinical features of patients in the neoadjuvant chemotherapy (NAC) cohort (n=124)

Factor	N	Percentage (%)
Age (years)		
⩽ 45	56	45.2
> 45	68	54.8
Menstruation status		
Premenopause	69	55.6
Menopause	55	44.4
cTumor size		
⩽ 4cm	86	69.4
> 4cm	38	30.6
cLymph node		
Negative	45	36.3
Positive	79	63.7
Histological grade		
I-II	67	54.0
III	57	46.0
Molecular subtype		
HR^+ $HER2^-$	58	46.8
HR^+ $HER2^+$	30	24.2
HR^- $HER2^+$	19	15.3
HR^- $HER2^-$	17	13.7
Trastuzumab		
No	75	60.5
Yes	49	39.5

The criteria to determine menopause include any of the following: a) Prior bilateral oophorectomy; b) Age ≥60 y; c) Age <60 y and amenorrheic for 12 or more months in the absence of chemotherapy, tamoxifen, toremifene, or ovarian suppression and follicle-stimulating hormone (FSH) and estradiol in the postmenopausal range; d) If taking tamoxifen or toremifene, and age <60 y, then FSH and plasma estradiol level in postmenopausal ranges. **Abbreviations:** cTumor size, clinical tumor size before NAC; cLymph node, clinical lymph node metastasis before NAC; HR, hormone receptor; HER2, human epidermal growth factor receptor 2.

Supplementary Table 2. Clinical features of patients in the treatment-naive cohort (n=388)

Factor	N	Percentage (%)
Age (years)		
≤ 45	134	34.5
> 45	254	65.5
Menstruation status		
Premenopause	197	50.8
Menopause	191	49.2
Tumor size		
≤ 2cm	217	55.9
> 2cm	171	44.1
Lymph node		
Negative	215	55.4
Positive	173	44.6
Histological grade		
I-II	233	60.1
III	155	39.9
Molecular subtype		
HR^+ $HER2^-$	216	55.7
HR^+ $HER2^+$	78	20.1
HR^- $HER2^+$	38	9.8
HR^- $HER2^-$	56	14.4

The criteria to determine menopause are same as Supplementary Table 1. **Abbreviations:** HR, hormone receptor; HER2, human epidermal growth factor receptor 2.
