# Supplementary material for: Niche stiffness sustains cancer stemness via TAZ and NANOG phase separation
Source: Nat Commun. 2023 Jan 16;14:238. doi: 10.1038/s41467-023-35856-y (PMC9842735; doi:10.1038/s41467-023-35856-y)
Supplement: Supplementary file 2 — Reporting Summary [file 41467_2023_35856_MOESM2_ESM.pdf]

nature portfolio

Corresponding author(s): Qiyi Zhao
Jian-You Liao
Shicheng Su

Last updated by author(s): Dec 5, 2022

Reporting Summary

Nature Portfolio wishes to improve the reproducibility of the work that we publish. This form provides structure for consistency and transparency in reporting. For further information on Nature Portfolio policies, see our Editorial Policies and the Editorial Policy Checklist.

Statistics

For all statistical analyses, confirm that the following items are present in the figure legend, table legend, main text, or Methods section.

n/a	Confirmed	
☐	☒	The exact sample size (n) for each experimental group/condition, given as a discrete number and unit of measurement
☐	☒	A statement on whether measurements were taken from distinct samples or whether the same sample was measured repeatedly
☐	☒	The statistical test(s) used AND whether they are one- or two-sided<br>*Only common tests should be described solely by name; describe more complex techniques in the Methods section.*
☒	☐	A description of all covariates tested
☐	☒	A description of any assumptions or corrections, such as tests of normality and adjustment for multiple comparisons
☐	☒	A full description of the statistical parameters including central tendency (e.g. means) or other basic estimates (e.g. regression coefficient) AND variation (e.g. standard deviation) or associated estimates of uncertainty (e.g. confidence intervals)
☐	☒	For null hypothesis testing, the test statistic (e.g. F, t, r) with confidence intervals, effect sizes, degrees of freedom and P value noted<br>*Give P values as exact values whenever suitable.*
☒	☐	For Bayesian analysis, information on the choice of priors and Markov chain Monte Carlo settings
☒	☐	For hierarchical and complex designs, identification of the appropriate level for tests and full reporting of outcomes
☐	☒	Estimates of effect sizes (e.g. Cohen's d, Pearson's r), indicating how they were calculated

Our web collection on statistics for biologists contains articles on many of the points above.

Software and code

Policy information about availability of computer code

Data collection	Fluorescent images: Laser scanning confocal microscopy (C2, Nikon; LSM880, and LSM980, Carl Zeiss; Dragonfly Spinning Disc Confocal, Andor Technology)<br>Flow cytometry: CytoFlex (Beckman Coulter)<br>Ultrasound elastography: HV-900 (Hitachi Medical).<br>Atomic force microscopy map: Atomic force microscopy (MFP3D-BIO, Asylum Research).
Data analysis	Data representation, Kaplan-Meier prognostic analysis and statistical analysis: GraphPad Prism 9 (GraphPad Software); SPSS (version 23, IBM Analytics); X-tile software (v3.6.1)<br>Fluorescent images: Imaris version 9.0 software (Bitplane); ZEN 2012 software (Carl Zeiss)<br>Atomic force microscopy map: Igor Pro (v 6.37, Wavemetrics).<br>Flow cytometry: FlowJo (version10, Treestar).

For manuscripts utilizing custom algorithms or software that are central to the research but not yet described in published literature, software must be made available to editors and reviewers. We strongly encourage code deposition in a community repository (e.g. GitHub). See the Nature Portfolio guidelines for submitting code & software for further information.

Data

Policy information about availability of data

All manuscripts must include a data availability statement. This statement should provide the following information, where applicable:

- Accession codes, unique identifiers, or web links for publicly available datasets
- A description of any restrictions on data availability
- For clinical datasets or third party data, please ensure that the statement adheres to our policy

All the data supporting the findings of this study are available within the main text of this article and its Supplementary Information. Source data are provided with this paper.

Field-specific reporting

Please select the one below that is the best fit for your research. If you are not sure, read the appropriate sections before making your selection.

☒ Life sciences ☐ Behavioural & social sciences ☐ Ecological, evolutionary & environmental sciences

For a reference copy of the document with all sections, see nature.com/documents/nr-reporting-summary-flat.pdf

Life sciences study design

All studies must disclose on these points even when the disclosure is negative.

Sample size	For clinical sample analysis, in vitro experiments, and animal experiments sample size was determined based on pilot experiments and similar research reported in literature on breast cancer (Yang, et al, nature, 2020; Su et al, Cell, 2018). All experiments included at least 3 independent experiments. The number of independent experiment was indicated in each figure legends.
Data exclusions	No data was excluded.
Replication	For each experiments the number of biological independent animal/sample/patient is reported in the figure legend. In vitro studies are represented at least 3 independent reproducible studies. Animal studies represent at least 6 independent mice. Studies using human tumor slices represent reproducible observations from independent cohorts of breast cancer patients.
Randomization	Animals were allocated randomly to each treatment group, and animals in different treatment groups were exposed to the same environment. For in vitro experiment, the samples were prepared and treated in random order. As the retrospective nature of our study design, randomization was not performed in clinical patients.
Blinding	Blinding was used in clinical sample analysis, quantification, and animal experiments.<br>No blinding was performed for in vitro experiments, since experiments were performed by the same investigator. However, samples were prepared, treated, and analyzed by the same standard procedure. Key experiments were validated in independent experiments.

Reporting for specific materials, systems and methods

We require information from authors about some types of materials, experimental systems and methods used in many studies. Here, indicate whether each material, system or method listed is relevant to your study. If you are not sure if a list item applies to your research, read the appropriate section before selecting a response.

Materials & experimental systems

n/a	Involved in the study	
☐	☒	Antibodies
☐	☒	Eukaryotic cell lines
☒	☐	Palaeontology and archaeology
☐	☒	Animals and other organisms
☐	☒	Human research participants
☒	☐	Clinical data
☒	☐	Dual use research of concern

Methods

n/a	Involved in the study	
☒	☐	ChIP-seq
☐	☒	Flow cytometry
☒	☐	MRI-based neuroimaging

Antibodies

Antibodies used	APC-conjugated Annexin V, cat# 640920, Biolegend, FCM: 1:20<br>PE anti-human CD24 Antibody (clone: ML5), Cat# 311106, BioLegend, FCM: 1:20<br>APC anti-human CD44 Antibody (clone: ML5BJ18), Cat# 338806, BioLegend, FCM: 1:20<br>Mouse anti-human CK (clone: AE1/AE3), cat# ab27988, Abcam, IF: 1:200

Goat anti-human CK cat# ab219271, Abcam, IF: 1:200
Goat anti-human Aldehyde Dehydrogenase 1, cat# AF5869, R&D Systems, IF: 1:100
Rabbit anti-human TAZ (clone: E8E9G), cat# 83669, Cell Signaling Technology, IF:1:100, IB: 1:1000
Mouse anti-human TAZ (clone: CL0371), cat# ab242313, Abcam, IF:1:100
Rabbit anti-human TAZ (clone: E9J5A), cat# 72804, Cell Signaling Technology, CHIP: 1:50
Goat anti-human OCT4, cat# AF1759, R&D Systems, IF: 1:100
Rabbit anti-human OCT4, cat# ab19857, Abcam, IB: 1:1000
Rabbit anti-human SOX2 (clone: D6D9), cat# 3579, Cell Signaling Technology, IF: 1:100 IB: 1:1000
Mouse anti-human NANOG (clone: 1E6C4), cat# 4893, Cell Signaling Technology, IF: 1:100 IB: 1:1000
Rabbit anti-human NANOG (clone: EPR2027(2)), ab109250, cat# ab109250, Abcam, IB: 1:1000
Rabbit anti-human NANOG, cat# 3580, Cell Signaling Technology, CHIP: 1:25
Anti-Digoxigenin-fluorescein, cat# 11207741910, Roche, IF: 1:100
Rabbit anti-human Caspase-3, cat# 9662, Cell Signaling Technology, IB: 1:1000
Rabbit anti-human Cleaved caspase-3, cat# 9661, Cell Signaling Technology, IB: 1:1000
Rabbit anti-human PARP (clone: 46D11), cat# 9532, Cell Signaling Technology, IB: 1:1000
Rabbit anti-human Cleaved PARP (clone: D64E10), cat# #5625, Cell Signaling Technology, IB: 1:1000
Mouse anti-human GAPDH (clone: 1E6D9), cat# 60004-1-Ig, Proteintech, IB: 1:10000
Rabbit anti-human α-tubulin, cat# 11224-1-AP, Proteintech, IB: 1:10000
Donkey anti-Mouse IgG, Alexa Fluor 555, cat# A-31570, ThermoFisher Scientific, IF: 1:300
Donkey anti-Mouse IgG, Alexa Fluor 488, cat# A-21202, ThermoFisher Scientific, IF: 1:300
Donkey anti-Mouse, Alexa Fluor 647, cat# A-31571, ThermoFisher Scientific, IF: 1:300
Donkey anti-Goat IgG, Alexa Fluor 555, cat# A-21432, ThermoFisher Scientific, IF: 1:300
Donkey anti-Goat IgG, Alexa Fluor 488, cat# A-11055, ThermoFisher Scientific, IF: 1:300
Donkey anti-Goat IgG, Alexa Fluor 647, cat# A-21447, ThermoFisher Scientific, IF: 1:300
Donkey anti-Rabbit IgG, Alexa Fluor 555, cat# A-31572, ThermoFisher Scientific, IF: 1:300
Donkey anti-Rabbit, Alexa Fluor 488, cat# A-21206, ThermoFisher Scientific, IF: 1:300
Anti-rabbit IgG, HRP-linked Antibody, cat#7074, Cell Signaling Technology, IB: 1:3000
Anti-mouse IgG, HRP-linked Antibody, cat# 7076, Cell Signaling Technology, IB: 1:3000

Validation

All antibodies used in our study were validated on the manufacturer's website.
APC-conjugated Annexin V, cat# 640920, Biolegend
https://www.biolegend.com/en-us/products/apc-annexin-v-8144?Clone=
PE anti-human CD24 Antibody, Cat# 311106, BioLegend
https://www.biolegend.com/en-us/products/pe-anti-human-cd24-antibody-1805
APC anti-human CD44 Antibody, Cat# 338806, BioLegend
https://www.biolegend.com/en-us/products/apc-anti-human-cd44-antibody-5583
Cytokeratin, cat# ab27988, Abcam
https://www.abcam.cn/pan-cytokeratin-antibody-ae1ae3-ab27988.html
Cytokeratin, cat# ab219271, Abcam
https://www.abcam.cn/cytokeratin-18-antibody-ab219271.html
Aldehyde Dehydrogenase 1, cat# AF5869, R&D
https://www.rndsystems.com/cn/search?keywords=ALDH1
TAZ, cat# 83669, Cell Signaling Technology
https://www.cellsignal.cn/products/primary-antibodies/taz-e8e9g-rabbit-mab/83669?site-search-type=Products&Ns=productCitationsCount%7C1&N=4294956287&Ntt=taz&fromPage=plp
TAZ, cat# ab242313, Abcam
https://www.abcam.cn/taz-antibody-cl0371-ab242313.html
TAZ, cat# 72804, Cell Signaling Technology
https://www.cellsignal.cn/products/primary-antibodies/taz-e9j5a-xp-rabbit-mab/72804?site-search-type=Products&N=4294956287&Ntt=72804&fromPage=plp&_requestid=6132605
OCT4, cat# AF1759, R&D Systems
https://www.rndsystems.com/cn/products/human-mouse-oct-3-4-antibody_af1759
OCT4, cat# ab19857, Abcam
https://www.abcam.cn/oct4-antibody-ab19857.html
SOX2, cat# 3579, Cell Signaling Technology
https://www.cellsignal.cn/products/primary-antibodies/sox2-d6d9-xp-rabbit-mab/3579?site-search-type=Products&N=4294956287&Ntt=3579%2C&fromPage=plp&_requestid=4790761
NANOG, cat# 4893, Cell Signaling Technology
https://www.cellsignal.cn/products/primary-antibodies/nanog-1e6c4-mouse-mab/4893?site-search-type=Products&N=4294956287&Ntt=nanog&fromPage=plp
NANOG, cat# ab109250, Abcam
https://www.abcam.cn/nanog-antibody-epr20272-ab109250.html
NANOG, cat# 3580, Cell Signaling Technology
https://www.cellsignal.cn/products/primary-antibodies/nanog-antibody/3580?site-search-type=Products&N=4294956287&Ntt=3580&fromPage=plp&_requestid=6136214
Anti-Digoxigenin-fluorescein, cat# 11207741910, Roche
https://www.sigmaaldrich.com/HK/zh/product/roche/11207741910
Caspase-3, cat# 9662, Cell Signaling Technology
https://www.cellsignal.cn/products/primary-antibodies/caspase-3-antibody/9662?site-search-type=Products&N=4294956287&Ntt=caspase-3&fromPage=plp
Cleaved caspase-3, cat# 9661, Cell Signaling Technology
https://www.cellsignal.cn/products/primary-antibodies/cleaved-caspase-3-asp175-antibody/9661?site-search-type=Products&N=4294956287&Ntt=caspase-3&fromPage=plp
PARP, cat# 9532, Cell Signaling Technology
https://www.cellsignal.cn/products/primary-antibodies/parp-46d11-rabbit-mab/9532?site-search-

type=Products&Ns=productCitationsCount%7C1&N=4294956287&Ntt=parp&fromPage=plp
Cleaved PARP, cat# #5625, Cell Signaling Technology
https://www.cellsignal.cn/products/primary-antibodies/cleaved-parp-asp214-d64e10-xp-rabbit-mab/5625?site-search-type=Products&Ns=productCitationsCount%7C1&N=4294956287&Ntt=parp&fromPage=plp
GAPDH, cat# 60004-1-Ig, Proteintech
https://www.ptgcn.com/products/GAPDH-Antibody-60004-1-Ig.htm
α-tubulin, cat# 11224-1-AP, Proteintech
https://www.ptgcn.com/products/TUBA1B-Antibody-11224-1-AP.htm
Donkey anti-Mouse IgG, Alexa Fluor 555, cat# A-31570, ThermoFisher Scientific
https://www.thermofisher.cn/cn/zh/antibody/product/Donkey-anti-Mouse-IgG-H-L-Highly-Cross-Adsorbed-Secondary-Antibody-Polyclonal/A-31570
Donkey anti-Mouse IgG, Alexa Fluor 488, cat# A-21202, ThermoFisher Scientific
https://www.thermofisher.cn/cn/zh/antibody/product/Donkey-anti-Mouse-IgG-H-L-Highly-Cross-Adsorbed-Secondary-Antibody-Polyclonal/A-21202
Donkey anti-Mouse, Alexa Fluor 647, cat# A-31571, ThermoFisher Scientific
https://www.thermofisher.com/antibody/product/Donkey-anti-Mouse-IgG-H-L-Highly-Cross-Adsorbed-Secondary-Antibody-Polyclonal/A-31571
Donkey anti-Goat IgG, Alexa Fluor 555, cat# A-21432, ThermoFisher Scientific
https://www.thermofisher.cn/cn/zh/antibody/product/Donkey-anti-Goat-IgG-H-L-Cross-Adsorbed-Secondary-Antibody-Polyclonal/A-21432
Donkey anti-Goat IgG, Alexa Fluor 488, cat# A-11055, ThermoFisher Scientific
https://www.thermofisher.cn/cn/zh/antibody/product/Donkey-anti-Goat-IgG-H-L-Cross-Adsorbed-Secondary-Antibody-Polyclonal/A-11055
Donkey anti-Goat IgG, Alexa Fluor 647, cat# A-21447, ThermoFisher Scientific
https://www.thermofisher.com/antibody/product/Donkey-anti-Goat-IgG-H-L-Cross-Adsorbed-Secondary-Antibody-Polyclonal/A-21447
Donkey anti-Rabbit IgG, Alexa Fluor 555, cat# A-31572, ThermoFisher Scientific
https://www.thermofisher.com/antibody/product/Donkey-anti-Rabbit-IgG-H-L-Highly-Cross-Adsorbed-Secondary-Antibody-Polyclonal/A-31572
Donkey anti-Rabbit, Alexa Fluor 488, cat# A-21206, ThermoFisher Scientific
https://www.thermofisher.com/antibody/product/Rabbit-IgG-H-L-Secondary-Antibody-Polyclonal/A-21206
Anti-rabbit IgG, HRP-linked Antibody, cat#7074, Cell Signaling Technology
https://www.cellsignal.cn/products/secondary-antibodies/anti-rabbit-igg-hrp-linked-antibody/7074?site-search-type=Products&N=4294956287&Ntt=7074&fromPage=plp&_requestid=4792912
Anti-mouse IgG, HRP-linked Antibody, cat# 7076, Cell Signaling Technology
https://www.cellsignal.cn/products/secondary-antibodies/anti-mouse-igg-hrp-linked-antibody/7076

Eukaryotic cell lines

Policy information about cell lines

Cell line source(s)	MCF-7 (cat# HTB-22), BT-474 (cat# HTB-20) and HEK293T (cat# CRL-3216) cell lines were obtained from the American Type Culture Collection.
Authentication	The cell lines have been authenticated by short tandem repeat profiling.
Mycoplasma contamination	Cell lines were all tested negative for mycoplasma contamination.
Commonly misidentified lines (See ICLAC register)	No commonly misidentified lines were used in this study.

Animals and other organisms

Policy information about studies involving animals; ARRIVE guidelines recommended for reporting animal research

Laboratory animals	Six-week-old female wild-type NOD/SCID mice were purchased from the Animal Experiment Center of Sun Yat-Sen University. All mice were maintained at the Animal Experiment Center of Sun Yat-Sen University under the 14-hour lights on/10-hour lights off cycle and at 22°C and 60% humidity.
Wild animals	The study did not involve wild animals.
Field-collected samples	The study did not involve samples collected from the field.
Ethics oversight	All animal experiments were approved by the Animal Care and Use Committee of Sun Yat-Sen University.

Note that full information on the approval of the study protocol must also be provided in the manuscript.

Human research participants

Policy information about studies involving human research participants

Population characteristics	This study included a total of 563 female adult invasive breast cancer patients enrolled at the Sun Yat-Sen Memorial Hospital, Sun Yat-Sen University (Guangzhou, China) between 2009 and 2022. Among these cases, a cohort consisting of 124 invasive

	breast carcinoma patients (Supplementary Table 1) received NAC. In addition, immunofluorescence staining for TAZ and NANOG was performed in 22 female patients and proximity ligation assay was performed in 29 cases who underwent neoadjuvant therapy at the Sun Yat-Sen Memorial between 2020 and 2022. Paired biopsy samples and surgically resected samples were collected from the same patients before and after neoadjuvant chemotherapy. Imaging examinations (breast ultrasound and magnetic resonance imaging) were applied to evaluate therapeutic effects according to the Response Evaluation Criteria in Solid Tumors (RECIST) guideline (version 1.1). Another cohort of 388 chemotherapy-naive invasive breast carcinoma samples (Supplementary Table 2) from Sun Yat-Sen Memorial Hospital, Sun Yat-Sen University (Guangzhou, China) between 2009 and 2022 were included for Kaplan-Meier survival analysis.
Recruitment	All samples were collected from patients with informed consent, and all related procedures were performed with the approval of the internal review and ethics boards of Sun Yat-Sen Memorial Hospital. No self-selection bias in recruitment.
Ethics oversight	All the related procedures were performed with the approval of the internal review and ethics boards of Sun Yat-Sen Memorial Hospital.

Note that full information on the approval of the study protocol must also be provided in the manuscript.

Flow Cytometry

Plots

Confirm that:

☒ The axis labels state the marker and fluorochrome used (e.g. CD4-FITC).

☒ The axis scales are clearly visible. Include numbers along axes only for bottom left plot of group (a 'group' is an analysis of identical markers).

☒ All plots are contour plots with outliers or pseudocolor plots.

☒ A numerical value for number of cells or percentage (with statistics) is provided.

Methodology

Sample preparation	MCF-7 and BT-474 cells cultured on hydrogels for 2 weeks were treated with chemotherapeutic agents (docetaxel or cisplatin) for 24 h. Then cells were dissociated by 0.25% trypsin-EDTA and harvested by centrifugation 1000 rpm for 5 min. To detect apoptotic cells, MCF-7 or BT-474 cells (1× 105 per sample) were incubated with 100 μL binding buffer (Biolegend) containing 5 μL APC-conjugated Annexin V (1:20, cat# 640920, Biolegend) for 15min at room temperature. After incubation, the cells were washed and resuspended in binding buffer (200 μL) containing 5 μL propidium iodide (PI, Biolegend) staining solution and detected in a flow cytometry immediately. Data were analyzed by FlowJo (Version 10, Treestar).<br>For cell surface marker staining and flow cytometric analysis, MCF-7 or BT-474 cells were resuspended in PBS containing 1% FBS and incubated with PE anti-human CD24 Antibody (Cat# 311106, BioLegend) and APC anti-human CD44 Antibody (Cat# 338806, BioLegend) for 30min at 4 °C. Afterwards, cells were washed with PBS. ALDH1 activity was measured using the ALDEFLUOR kit (Cat# 01700, Stem Cell Technologies) according to the manufacturer's instructions. Specimens were subsequently analyzed by a CytoFlex Flow cytometer (Beckman Coulter). Data were analyzed using FlowJo software (version10, Treestar).
Instrument	CytoFlex (Beckman Coulter)
Software	FlowJo (Version 10, Treestar)
Cell population abundance	Percentage of Annexin V+ cells, CD24lowCD44high cells, and ALDH1+ cells determined was by flow cytometry. Purity of the samples was >95%.
Gating strategy	The cells were gated by FSC/SSC gates. Gates indicating boundaries between "positive" and "negative" are according to the isotype staining . The sorting gate of the ALDH1+ cells was established using DEAB-treated cells as a reference. Expression of indicated proteins were checked on these populations as indicatated in the figures and figure legends.

☒ Tick this box to confirm that a figure exemplifying the gating strategy is provided in the Supplementary Information.
